# Ultrasound-assisted extraction optimization and validation of an HPLC-DAD method for the quantification of polyphenols in leaf extracts of *Cecropia* species

**DOI:** 10.1038/s41598-018-37607-2

**Published:** 2019-02-14

**Authors:** Andrés Rivera-Mondragón, Géraldine Broeckx, Sebastiaan Bijttebier, Tania Naessens, Erik Fransen, Filip Kiekens, Catherina Caballero-George, Yvan Vander Heyden, Sandra Apers, Luc Pieters, Kenn Foubert

**Affiliations:** 1 Natural Products & Food Research and Analysis (NatuRA), Department of Pharmaceutical Sciences, University of Antwerp, Universiteitsplein 1, 2610 Antwerp, Belgium; 2 Laboratory of Pharmaceutical Technology and Biopharmacy, Department of Pharmaceutical Sciences, University of Antwerp, Universiteitsplein 1, 2610 Antwerp, Belgium; 3 StatUa Center for Statistics, University of Antwerp, Prinsstraat 13, 2000 Antwerp, Belgium; 4 Centre of Innovation and Technology Transfer, Institute of Scientific Research and High Technology Services (INDICASAT-AIP), Building 208, City of Knowledge, Panama, Republic of Panama; 5 Department of Analytical Chemistry, Applied Chemometrics and Molecular Modelling, Vrije Universiteit Brussel–VUB, Laarbeeklaan 103, B-1090 Brussels, Belgium

## Abstract

*Cecropia* species are traditionally used in Latin American folk medicine and are available as food supplements with little information warranting their quality. The optimum conditions for the extraction of chlorogenic acid (CA), total flavonoids (TF) and flavonolignans (FL) from leaves of *Cecropia* species were determined using a fractional factorial design (FFD) and a central composite design (CCD). A reversed-phase high-performance liquid chromatographic method coupled to a diode array detector (HPLC-DAD) was validated for the quantification of CA, TF and FL, following the ICH guidelines. Quantitative and Principal Component Analysis (PCA) was also performed. The extraction-optimization methodology enabled us developing an appropriate extraction process with a time-efficient execution of experiments. The experimental values agreed with those predicted, thus indicating suitability of the proposed model. The validation parameters for all chemical markers of the quantification method were satisfactory. The results revealed that the method had excellent selectivity, linearity, precision (repeatability and intermediate precision were below than 2 and 5%, respectively) and accuracy (98–102%). The limits of detection and quantification were at nanogram per milliliter (ng/mL) level. In conclusion, the simultaneous quantification of chemical markers using the proposed method is an appropriate approach for species discrimination and quality evaluation of *Cecropia* sp.

## Introduction

Plant species, popularly known as ‘yarumo’, ‘guarumo’, ‘guarumbo’, ‘embauba’, ‘ambay’ and ‘trumped tree’, belong to the genus *Cecropia* Loefl. (Urticaceae), which comprises 61 species and are distributed across the tropical and subtropical rainforest from Mexico over Central to South America below an altitude of 2600 m^1,2^.

Some *Cecropia* species, such as *C*. *obtusifolia* Bertol., *C*. *peltata* L., *C*. *pachystachya* Trécul, *C*. *hololeuca* Miq. and *C*. *glaziovii* Snethl. are traditionally used in Latin American folk medicine for the treatment of a variety of diseases, like diabetes^3^, hypertension^4^ and inflammation^5^. Additionally, they have been reported for their wound healing^6^ and antimalarial^7^ activities. These pharmacological properties have been correlated to their content of phenolic compounds, such as phenolic acids, flavonoids and terpenoids^5,8,9^.

A number of studies on *Cecropia* species suggested the relationship between the major compounds of the leaf extracts (relative concentration above 5 µg/g), chlorogenic acid and glycosyl flavonoids (orientin, isoorientin, vitexin and rutin) and the anti-diabetic^10^, anti-inflammatory^11^ and anti-hypertensive^12^ activities. Recently, a detailed phytochemical study on *Cecropia* species collected in Panama, also revealed the presence of flavonolignans in *C*. *obtusifolia*, *C*. *peltata*, *C*. *insignis* for the first time (Rivera-Mondragón *et al*.^2^ Submitted).

Taking this into consideration, chlorogenic acid, total flavonoids and flavonolignans have been selected as suitable chemical markers for the qualitative evaluation of leaves of plants of the genus *Cecropia*^2^. The relationship between these phenolic compounds and their pharmacological properties produces a big interest in the development and validation of an adequate analytical method, which will be useful for the quantification of herbal products containing these compounds. Additionally, the optimization of their extraction is of interest, since the chromatographic analysis only allows observing compounds properly extracted from the plant material.

A literature survey revealed that very little was published on developing and validating analytical methods for the quantitative determination of the main phenolic compounds in *Cecropia* species. For instance, a spectrophotometric colorimetric method (aluminium chloride) and HPLC-DAD methods have been developed for the determination of the seasonal variation of total flavonoids, isoorientin and isovitexin contents in dry leaves of *C. glaziovii* leaves^8,13^. In other investigations, LC methods have been described for the chemical standardization of *C. glaziovii*^14–16^, to isolate C-glycosyl flavones in *C. lyratiloba*^17^, to chemically fingerprint and quantify aqueous, methanolic and ethyl-acetate extracts of *C. pacystachya*^11,18,19^, and to evaluate the phytochemical composition of ethanolic extracts of *C. obtusifolia* and *C. peltata*^20^. Furthermore, two validated HPLC-DAD and UPLC-DAD methods for the quantification of chlorogenic acid and main flavone *C-*glycosides in leaves of *C. glaziovii*, *C. pachystachya* and *C. hololeuca* have been reported^21,22^.

However, to our knowledge, optimal extraction conditions and a complete validated method for the simultaneous identification and quantification of chlorogenic acid, total flavonoids and flavonolignans in *Cecropia* leaves have not been reported yet in peer-reviewed literature.

In this paper, we report a fully validated, sensitive and specific HPLC-DAD method for the quantification of CA, TF and FL in different authentic and commercial products of leaves of *Cecropia* species, collected in different regions of the Republic of Panama and obtained from the market. Furthermore, the extraction of the compounds of interest was optimized.

## Results and Discussion

### HPLC-DAD analysis of CA, TF and FL

Liquid chromatography coupled with mass spectrometry (LC-MS) offers better sensitivity and selectivity for compound identification in comparison with UV detection. However, the high cost of this equipment together with the absence of primary standards may be a limiting factor for low-budget analytical laboratories. On the other hand, DAD is inexpensive, broadly applied to natural product analysis, has a reliable and reproducible performance, and provides the possibility of online collection of UV spectra, inducing a viable added value for quantitative analysis^23^. Hence, we present an HPLC-DAD method for the routine analysis of CA, TF and FL in samples *Cecropia* species.

**Figure 1:**
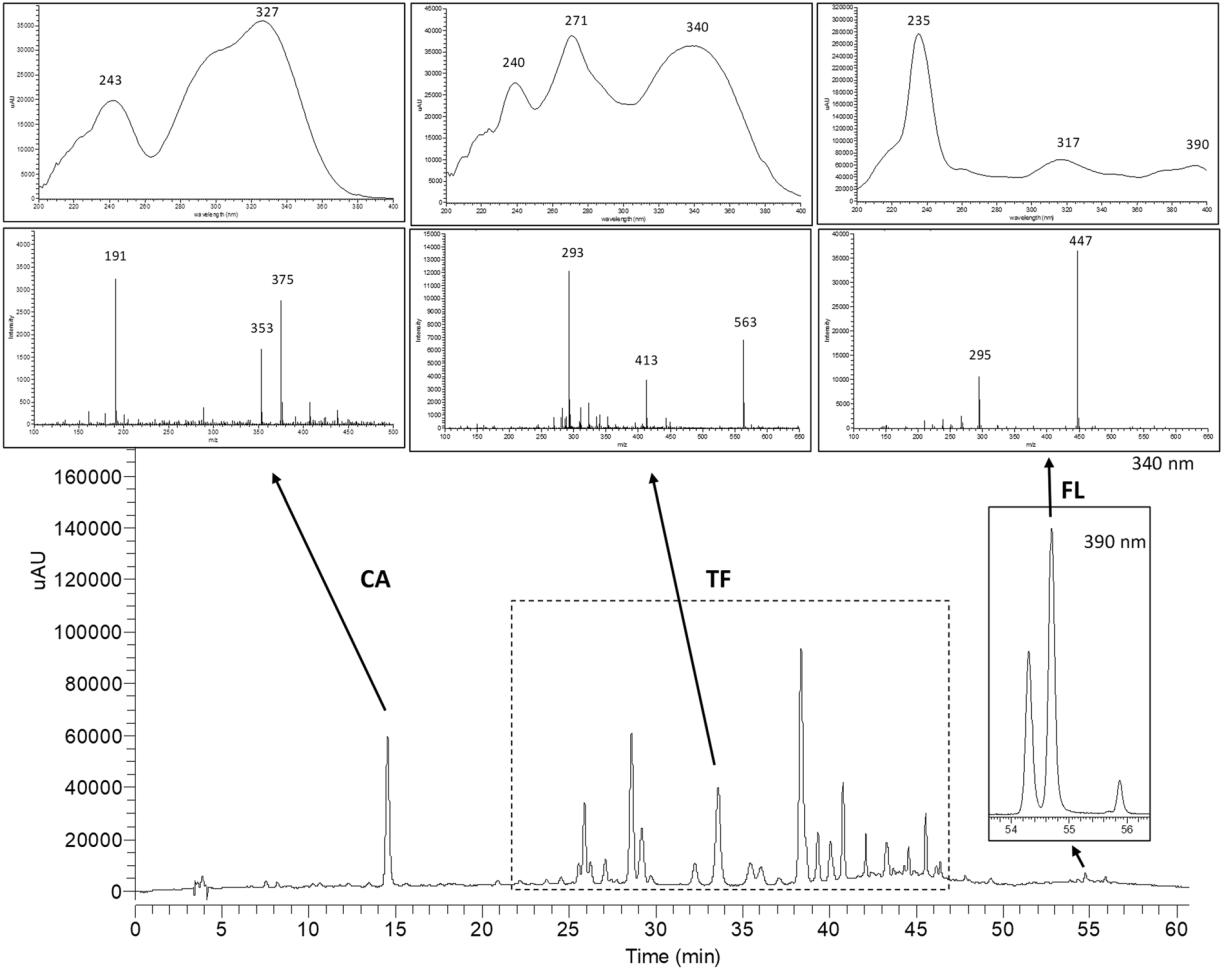
HPLC chromatogram of leaf extracts from a *Cecropia* species mixture measured on DA detector at 340 and 390 nm, and the observed characteristic UV and MS spectra of chlorogenic acid (CA), total flavonoids (TF) and flavonolignans (FL).

A representative HPLC-DAD profile of a leaf extract from a *Cecropia* species mixture is given in Fig. 1. Analysis of online UV spectra, obtained from peaks between 25–46 min, let us recognize typical UV absorption bands for flavonoids (Band I, λ max around 300–354 nm and Band II, λ max around 240–285 nm)^24^. As previously reported by our research group, flavone *C*-glycosides were predominately detected in *C. obtusifolia*, *C. peltata* and *C. insignis*; while quercetin *O*-glycosides were the main flavonoids described in *C. hispidissima* (Rivera-Mondragón *et al*., 2018. Submitted). Considering this, we decided to express *O*-glycosyl flavonols and *C*-glycosyl flavones as rutin (RU) and vitexin (VX) equivalents, respectively. FL such as mururin A and vaccinin A are not available as reference standard on the market. Therefore, VX was chosen as a secondary analytical standard for the quantification of these compounds.

### Extraction optimization

Variables with major effects over the extraction yields were optimized. First, ultrasound assisted extraction (UAE) was selected as the most appropriate extraction method due to its efficacy offering a great reduction of extraction time and low environmental impact^25–27^. Following, mixtures of water: methanol and acetone were chosen as extraction solvents, since these solvents have widely described as the most suitable systems for polyphenol extraction from plant material^28,29^.

The screening and optimization steps of TF, CA and FL from *Cecropia* species leaves were conducted by fractional factorial (FFD) and central composite (CCD) designs, respectively.

#### Screening (FFD)

Seven factors were chosen to be screened: methanol fraction (%, v/v) (A), extraction time (min) (B), number of extractions with methanol (C), extraction temperature (°C) (D), mass/solvent ratio (w/v) (E), number of extractions with acetone (F) and particle size of the plant material (µm) (G). The FFD, including the fraction levels and the experimental values of TF, CA and FL, is presented in the supplementary material (Table S1). The estimated effect of each factor on the responses and the corresponding critical effects are shown in Table 1. Those factors with effects below the critical effect (*E*_*critical*_) were considered statistically not significant factors affecting the yield of phenolic extraction.

**Table 1:** Factor effects from screening the *Cecropia* species leaves extraction process by FFD.

Factor	Effects		
	TF	CA	FL
A	−92.88*	−19.99*	1.12*
B	38.54	9.66	−0.05
C	76.98	24.70*	0.94*
D	149.19*	31.99*	1.55*
E	39.50	18.37*	0.40
F	12.65	0.58	0.85
G	−73.20	−13.01*	−0.03
A × B	−22.59	−6.84	−0.08
A × C	−14.37	1.38	−0.20
A × D	50.12	4.40	−0.44
A × E	−8.85	−3.20	−0.31
A × F	−24.34	−5.63	−0.08
A × G	−47.06	−3.64	−0.02
B × D	−27.05	1.27	−0.45
E_*critical*_	84.54	10.97	0.47

Factors: (A) methanol fraction (%, v/v), (B) extraction time (min), (C) number of methanol extractions, (D) temperature (°C), (E) mass:solvent ratio (w/v), (F) number of acetone extractions, (G) particle size of the plant material (µm). Responses: sum of peak areas of total flavonoids (TF), chlorogenic acid (CA) and flavonolignans (FL). The responses are represented as mean; SD are not given. *Significant effects.

TF, CA and FL extraction yields were significantly affected by the methanol fraction (A) and the extraction temperature (D). CA and FL contents were also significantly affected by the number of extractions (C). Additionally, CA content was significantly affected by mass/solvent ratio (E) and particle size (G). For the optimization step, we selected the three factors with the major effects on TF, CA and FL yields: methanol fraction (A), number of extractions with methanol (C) and extraction temperature (D). The number of extractions (C), a discrete variable, was fixed at three (3) extractions. The less significant factors were mass/solvent ratio (E) and particle size (G), and were established as 1:50 (m/v) and ≤125 µm, respectively, since those are the best tested to obtain the highest CA yield. In addition, one (1) acetone extraction (F) was set, because it has a significant positive effect on FL peak area. Extraction time (B) was set at 30 min, since it has no significant effect neither on CA, TF or FL.

#### Optimization (CCD)

A response surface methodology (RSM), using a CCD, was employed to optimize the effects of the methanol fraction (*X*_1_) and extraction temperature (*X*_2_) on the extraction yields of TF (*Y*_1_), CA (*Y*_2_) and FL (*Y*_3_). The other variables were set as mentioned in the previous section (*Screening FFD*). Supplementary Tables S2 and S3 show the independent variables and their levels used for this experiment, and the actual design and the experimental data for the response variables, respectively. Second-order polynomial equations were built to describe the relationship between *X*_1_ and *X*_2_ (Table 2).

**Table 2:** Quadratic polynomial equations for the three responses.

Responses	Equations
TF	*Y*_1_ = 1737.8 − 106.1*X*_1_ + 43.8*X*_2_–86.9*X*_1_^2^ − 6.8*X*_2_^2^ + 47.3*X*_1_*X*_2_
CA	*Y*_2_ = 340.7 − 22.0*X*_1_ + 14.6*X*_2_ − 23.2*X*_1_^2^ − 2.4*X*_2_^2^ + 1.8*X*_1_*X*_2_
FL	*Y*_3_ = 14.0 + 0.35*X*_1_ + 0.33*X*_2_ − 0.63*X*_1_^2^ − 0.27*X*_2_^2^ − 0.16*X*_1_*X*_2_

*Y*_1_, *Y*_2_ and *Y*_3_ are the responses. *X*_1_ and *X*_2_ are the independent variables, methanol fraction (%, v/v) and temperature extraction (°C), respectively.

The analysis of variance (ANOVA) for the quadratic polynomial models developed for the response variables indicated that the linear effects of *X*_1_ and *X*_2_ were found to be significant (p < 0.05) for TF, CA and FL extraction (Table 3). Besides, *X*_1_^2^ is significant (p < 0.05) for all response variables, while *X*_2_^2^ was not significant (p > 0.05) for any response. On the other hand, the interaction effect of *X*_1_*X*_2_ is only significant (p < 0.05) for TF.

**Table 3:** A *t*-test for the quadratic polynomial models developed for the response variables: total flavonoids (TF), chlorogenic acid (CA) and flavonolignans (FL) of *Cecropia* sp. mixture.

Term	TF (*Y*_1_)			CA (*Y*_2_)			FL (*Y*_3_)		
	Coefficient	Standard error	*p*-Value	Coefficient	Standard error	*p*-Value	Coefficient	Standard error	*p*-Value
Model			<0.0001			<0.0001			0.0028
Intercept	1737.8	29.3	3.48E-16	340.7	3.64	1.50E-18	13.96	0.302	6.82E-15
**Linear terms**									
*X*_1_	−106.1	10.4	2.75E-07	−22.0	1.29	8.59E-10	0.350	0.107	0.0066
*X*_2_	43.8	10.4	0.0012	14.6	1.29	8.92E-08	0.329	0.107	0.0094
**Quadratic terms**									
*X*_1_^2^	−86.9	17.2	0.0003	−23.2	2.14	1.47E-07	−0.635	0.177	0.0038
*X*_2_^2^	−6.8	17.2	0.6980	−2.40	2.14	0.2830	−0.273	0.177	0.1484
**Interaction**									
*X*_1_ *X*_2_	47.3	14.6	0.0072	1.81	1.82	0.3400	−0.161	0.151	0.3060

**Figure 2:**
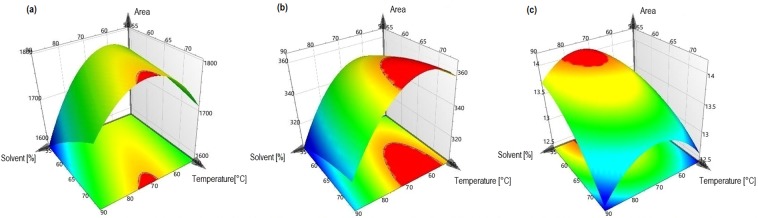
Response surface plots demonstrating the influence of the methanol fraction (%, v/v) and the extraction temperature (°C) on the peak areas of TF (A), CA (B) and FL (C).

In order to visualize the factor and interaction effects on the extraction efficiency, the three-dimensional response surface plots were generated as a function of *X*_1_ and *X*_2_ as shown in Fig. 2. In general, the peak areas of TF, CA and FL increased within a range of methanol fraction of 70–75% (v/v), 55–72% (v/v) and 70–80% (v/v), respectively. However, a methanol fraction below or above this ranges appeared to decrease the extraction yields of these compounds. Furthermore, the highest TF, CA and FL extraction yields were observed with extraction temperature ranges from 70–75 °C, 65–75 °C and 55–65 °C, respectively. FL extraction efficiency was negatively affected when the extraction temperature was above 65 °C. For TF and CA the extraction yields were only affected to a limited extend by the temperature.

#### Verification of the predicted optimal extraction conditions

The optimal *X*_1_ and *X*_2_ variables were determined by maximizing the responses using MODDE Pro Software. In order to confirm the reliability of the mathematical model, experimental extractions were performed under conditions selected as optimal: a methanol fraction of 70% (v/v), a extraction temperature of 64 °C, 30 min of extraction time, three (3) repeated extractions with methanol and one (1) with acetone, a mass/solvent ratio of 1:50, w/v, and particle size of ≤125 µm. The predicted and experimental responses demonstrated no significant differences (t-test, p > 0.05) and the difference between the predicted and experimental values were less than 2.0%, indicating an appropriate fitness of the predicted model (See Table 4).

**Table 4:** Experimental and predicted values of TF, CA and FL at optimal conditions.

Responses	Optimal extraction conditions		Maximun value		
	*X*_1_ (%, v/v)	*X*_2_ (°C)	Predicted	Experimental (n = 3)	% Difference (CV)
TF	70	64	1786.0 ± 29.3^a^	1736.3 ± 7.9^a^	0.94
CA			356.6 ± 3.6^b^	354.4 ± 3.2^b^	0.85
FL			13.9 ± 0.3^c^	14.1 ± 0.3^c^	1.79

All the values are means ± standard deviations and those sharing the same superscript letter in the same row are not significantly different from each other (p > 0.05).

**Figure 3:**
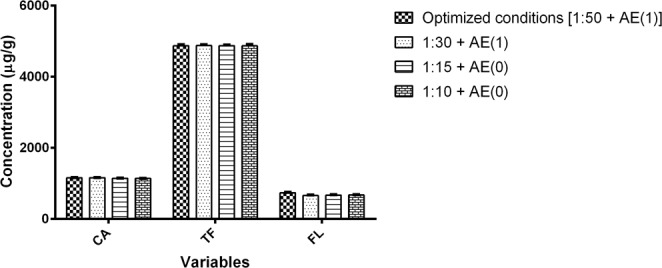
Evaluation of simultaneous variation of mass solvent ratio (1:30, 1:15 and 1:10) and number of acetone extractions (AE) compared to the optimized method conditions situation [1:50 + AE(1)].

In order to develop a less time- and solvent-consuming method, the robustness of the yield, was determined from different combinations of mass/solvent ratio (1:30, 1:15 and 1:10) and number of acetone extractions (1 and 0) (Fig. 3). One-way ANOVA followed by a Bonferroni post hoc test revealed not significant differences in CA, TF and FL contents (p >0.05) in comparison with the yield at the optimized conditions. Results show that mass/solvent ratio and number of acetone extractions may be changed (reduced) without compromising the analytes extraction. During the validation of the method, the mass/solvent ratio was set at 1:30, while the extraction with acetone-extraction was eliminated from the procedure.

### Validation of the HPLC-DAD method

#### Specificity

**Figure 4:**
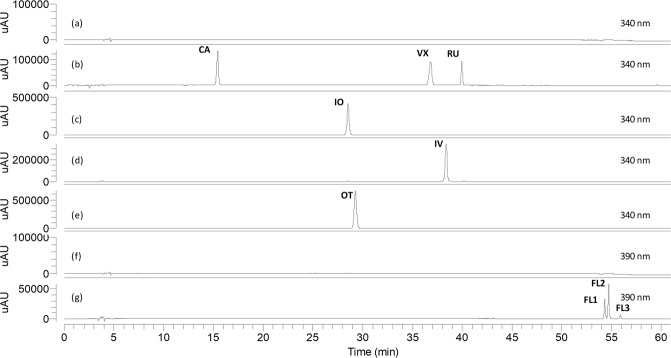
HPLC chromatograms of blank solution (**a,f**), chlorogenic acid (CA), vitexin (VX) and rutin (RU) (**b**), isoorientin (IO) (**c**), isovitexin (IV) (**d**), orientin (OT) (**e**), flavonolignan 1 (FL1), flavonolignan 2 (FL2) and flavonolignan 3 (FL3) (**g**). Chromatograms (**a–e**) were obtained at 340 nm, (**f**,**g**) at 390 nm.

**Figure 5:**
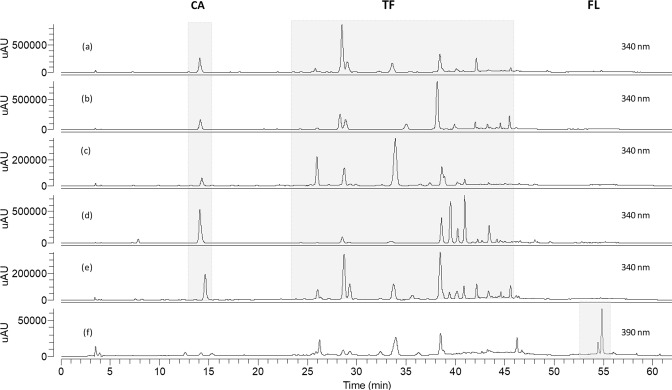
HPLC chromatograms of authentic samples: *C. obtusifolia* CO-5 (**a**), *C. peltata* CP-1 (**b**), *C. insingnis* CI-1 (**c**), *C. hispidissima* CH-1 (**d**), mixture of *Cecropia* species (**e**), and *C. obtusifolia* CO-6 (**f**).

**Figure 6:**
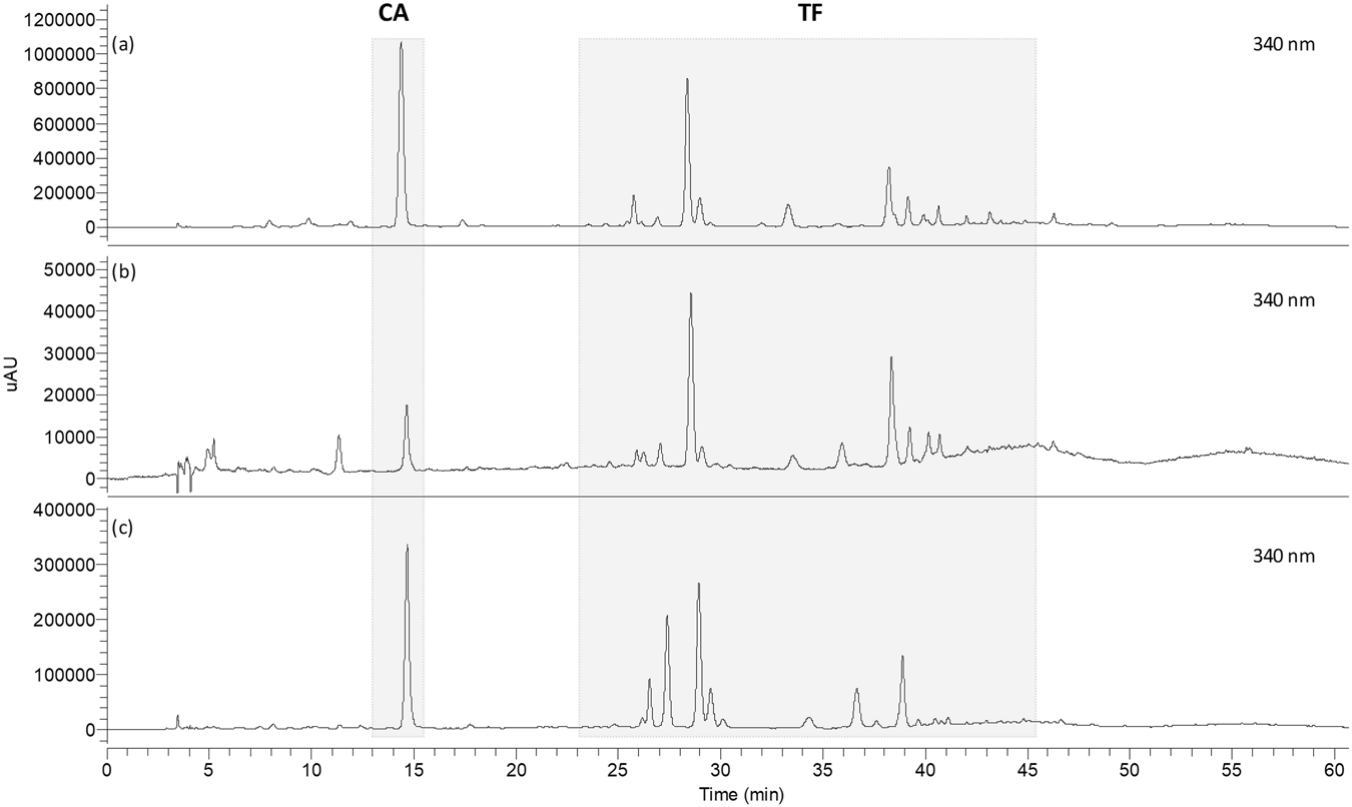
HPLC chromatograms of commercial samples: *C. obtusifolia* CO-C (**a**), *C. peltata* CP-C (**b**) and *C. hololeuca* CHO-C (**c**).

Blank solution, authentic standards and isolated compounds from *Cecropia* obtusifolia (FL1, FL2 and FL3) were analyzed under the analytical method conditions established here (Fig. 4). Authentic and commercial sample extracts from different species were analyzed as well in order to verify for possible interferences (Figs 5 and 6). Representative UV and MS spectra inspection of each analyte (Fig. 1), shows no relevant interferences at the regions (retention times) of interest. Despite some overlapping peaks were found (Fig. 5c), identified as flavonoids, the quantification of the total flavonoids is not interfered.

#### Linearity

Four curves were plotted at different levels (n ≥ 5) within an appropriate concentration range of CA, VX and RU, and the residuals were observed to be homoscedastic and with % RSD values below than 5% (Supplementary Fig. S2). As shown in Table 5, all curves have a linear response with r^2^ > 0.999, t-tests (N-2, p = 95%) demonstrated that intercepts were not significantly different from zero, while slopes were significantly different from zero. Additionally, 95% CI of intercepts include zero, which means that quantification analysis can be performed based on a single-point calibration approach. Moreover, Mandel’s fitting tests indicate that first-order calibration function (linear equation) provides a significantly (α = 0.01) better fit than second-order calibration (quadratic equation).

**Table 5:** Parameters of the calibration model for CA, VX and RU. Confidence interval (CI). Vitexin detected at 340 nm is represented as VX-1.

Analyte	Concentration range (µg/mL)	Equation	r^2^	95% CI of the intercept	LoD (ng/mL)	LoQ (ng/mL)
CA	0.16–50.30	y = 67.6x − 15.2	>0.99	−32.62–2.27	160.7	401.9
VX-1	0.16–211.70	y = 74.4x − 12.4	>0.99	−42.64–17.92	131.2	328.0
VX-2	1.63–352.83	y = 5.2x − 3.4	>0.99	−7.16–0.46	423.6	903.8
RU	1.49–194.64	y = 41.4x − 11.4	>0.99	−23.31–0.57	131.0	388.6

Vitexin detected at 390 nm as VX-2.

#### Precision

Repeatability (intra-day and intra-concentration level, n = 6) and intermediate precision (between 4 days, and three concentration levels, n = 6) were evaluated in order to assess the precision of the method. Overall, Cochran’s C test for homogeneity of variances (95% confidence level) indicated that variances between days and concentration levels are homogeneous (Supplementary Table S4). Values of % RSD for all parameters were below than 2.0% and 5.0% for repeatability and intermediate precision, respectively. In addition, all values did not exceed the levels calculated by the Horwitz equation and thus confirmed the expected performance of the method in terms of precision. Results are summarized in Table 6.

**Table 6:** Precision of the method for CA, TF and FL during 4 days and at three concentration levels.

Analyte	Relative standard deviation (% RSD)									
	Days (100%)				Concentration levels		Overall repeatability	Intermediate precision	Horwitz equation	
	1	2	3	4	50%	150%			RSD_r_	RSD_R_
CA	0.91	1.30	1.43	0.83	0.96	1.14	1.10	4.34	3.79	5.68
TF-1^a^	0.70	0.36	0.58	0.68	0.88	0.70	0.67	3.54	2.92	4.37
TF-2^b^	0.24	0.25	0.32	0.26	0.25	0.23	0.26	2.92	2.58	3.86
FL	1.19	1.39	1.86	0.99	0.91	1.46	1.32	4.77	4.23	6.34

n = 6, overall repeatability: intra days and levels, intermediate precision: inter days and levels. Overall variances were homogenous according Cochran’s C test (95% confidence level). ^a^TF expressed as VX equivalent (*C*. species mixture). ^b^TF expressed as rutin equivalent (*C*. *hispidissima*).

#### Accuracy

The accuracy was determined by means of a recovery experiment, adding known quantities of CA, VX and RU in three concentration levels (75, 100 and 125%). Fortified solutions were analyzed and the results were reported as percent recovery (%). Table 7 shows that accuracy data are in agreement with the acceptance criteria at all three concentrations levels since recovery values varied between 98 and 102%, 95% CI of the mean include 100%, and % RSD for each level concentration were lower or equal to % RSD_r_^30^.

**Table 7:** Accuracy of the method for CA, VX-1, VX-2 and RU determined by recovery (%). Confidence interval (CI). n = 3.

Analyte	Recovery (%)					
	75%	100%	125%	Mean	% RSD	95% CI
CA	101.41	100.10	99.96	100.49	0.99	99.73–101.25
VX-1^a^	99.77	99.55	100.21	99.84	0.57	99.40–100.28
VX-2^b^	100.40	99.02	100.06	99.83	0.90	99.13–100.52
RU	100.09	100.40	100.22	100.24	0.21	100.08–100.40

^a^Vitexin detected at 340 nm (VX-1). ^b^Vitexin detected at 390 nm (VX-2).

#### Limit of detection (LoD) and limit of quantification (LoQ)

LoD and LoQ were estimated based on the calibration curves of CA, VX and RU, constructed in a 1.5–50 µg/mL range (data not shown). Additionally, these estimates were subsequently validated by the independent analysis of six quantified samples (prepared by serial dilutions) in order to find concentration levels around LoD and LoQ. All compounds showed LoD and LoQ of the order of magnitude of ng/mL. CA, VX and RU showed lower concentrations of LoD and LoQ than those calculated by the mathematical method, except for VX detected at 390 nm, which displayed a higher LoD and a similar LoQ to those determined by its calibration curve (See supplementary material Table S5). This method is able to detect and quantify CA, TF (expressed as vitexin or rutin equivalent) and FL (expressed as vitexin equivalent) of *Cecropia* species in concentrations below than 0.5 µg/mL and 1.0 µg/mL, respectively (Table 5).

### Quantitative and multivariate analysis

Subsequent to the optimization of the extraction and the validation of the proposed analytical method, the contents of CA, TF and FL from fourteen authentic *Cecropia* leaves and three commercial products were assessed. The concentrations of phenolics found in *Cecropia* are shown in Table 8. It was observed that CA was present in all samples ranging from 78.7 ± 4.3 (CP-C) to 14724.5 (CO-7) (µg/g). Quantitative analysis of samples from Cerro Azul, Camino de Cruces and Cerro Campana showed higher CA concentrations collected during the rainy season (October, 2015) compared to those collected in the second dry period during the rainy season (known as First Canicula, July 2016); except for CO-7, which presented the highest CA content and was collected in Chiriqui (July, 2017) (See Supplemenatary Fig. S3).

**Table 8:** Concentrations of CA, TF and FL (µg/g) in authentic and commercial *Cecropia* leaf samples.

	Compounds	CO-1	CO-2	CO-3	CO-4	CO-5	CO-6	CO-7	CO-C	CP-1	CP-2
	***Phenolic acids***										
1	Chlorogenic acid	**1933.2 ± 36.9**	**1634.0 ± 40.7**	**243.6 ± 2.8**	**4238.1 ± 33.9**	**1393.3 ± 56.4**	**1091.5 ± 17.9**	**14724.5 ± 102.7**	**5612.6 ± 228.5**	**2217.8 ± 59.6**	**949.0 ± 35.0**
	***Flavonoids C-glycosides and di-C***,***O-glycosides***										
2	Luteolin *C*-hexoside-*O*-hexoside (1)	181.4 ± 4.0	277.6 ± 14.5	33.3 ± 1.7	<LOQ	<LOQ	40.2 ± 1.5	<LOQ	83.2 ± 0.4	<LOQ	<LOQ
3	Isoorientin-2′′-*O*-xyloside	455.7 ± 9.8	884.0 ± 11.1	196.9 ± 3.0	<LOQ	284.5 ± 6.3	69.7 ± 1.3	<LOQ	612.2 ± 9.7	<LOQ	<LOQ
4	Luteolin *C*-hexoside-*O*-hexoside (2)	65.7 ± 1.8	233.8 ± 3.3	<LOQ	<LOQ	<LOQ	366.8 ± 8.1	<LOQ	85.8 ± 3.2	<LOQ	<LOQ
5	Isoorientin-4′′-*O*-xyloside	105.1 ± 10.5	370.2 ± 11.5	<LOQ	<LOQ	<LOQ	<LOQ	<LOQ	204.2 ± 12.2	<LOQ	<LOQ
6	Isoorientin	384.6 ± 8.3	1077.7 ± 10.2	40.2 ± 3.3	6399.4 ± 25.5	4187.9 ± 119.8	160.9 ± 1.5	5177.7 ± 54.5	3661.7 ± 74.0	1796.2 ± 39.2	2127.9 ± 54.4
7	Orientin	<LOQ	<LOQ	<LOQ	1117.6 ± 9.7	1167.4 ± 35.5	<LOQ	4523.8 ± 44.9	<LOQ	1076.9 ± 13.6	749.3 ± 18.2
8	Isoorientin-2′′-*O*-rhamnoside	612.0 ± 17.0	241.2 ± 5.7	164.0 ± 5.0	<LOQ	<LOQ	129.8 ± 1.6	<LOQ	823.0 ± 20.1	<LOQ	<LOQ
9	Luteolin *C*-hexoside-*O*-pentoside	84.8 ± 3.7	145.1 ± 4.6	23.9 ± 4.0	<LOQ	<LOQ	<LOQ	<LOQ	119.7 ± 7.7	<LOQ	<LOQ
10	Isovitexin-2′′-*O*-glucoside	240.4 ± 6.7	465.3 ± 8.4	269.6 ± 2.4	<LOQ	112.1 ± 7.4	315.3 ± 8.4	<LOQ	118.3 ± 5.9	<LOQ	<LOQ
11	Isovitexin-2′′-*O*-xyloside	761.4 ± 19.7	1671.4 ± 26.2	1936.3 ± 11.4	<LOQ	1044.7 ± 36.3	2142.9 ± 71.9	<LOQ	787.1 ± 16.8	266.5 ± 3.1	410.0 ± 19.6
12	Vitexin	170.1 ± 5.9	<LOD	<LOQ	<LOQ	98.0 ± 1.3	<LOQ	295.2 ± 3.6	<LOQ	198.2 ± 2.1	457.4 ± 37.2
13	Apigenin *C*-hexoside-*O*-pentoside	156.2 ± 4.1	511.8 ± 6.5	80.6 ± 1.5	<LOQ	<LOQ	233.4 ± 3.0	<LOQ	138.8 ± 4.3	<LOQ	<LOQ
14	Diosmetin *C*-hexoside-*O*-pentoside	58.0 ± 1.0	134.1 ± 1.9	<LOQ	<LOQ	<LOQ	<LOQ	<LOQ	<LOQ	<LOQ	<LOQ
15	Isovitexin 2′′-*O*-rhamnoside	364.4 ± 8.5	424.8 ± 7.8	1057.6 ± 5.2	<LOQ	1376.9 ± 36.8	1356.7 ± 30.8	<LOQ	<LOQ	1107.2 ± 4.7	2338.1 ± 103.2
16	Apigenin *C*-hexoside-*O*-xyloside	177.7 ± 5.1	436.9 ± 5.2	287.5 ± 2.5	<LOQ	<LOQ	179.1 ± 4.7	<LOQ	<LOQ	<LOQ	<LOQ
17	Isovitexin	<LOQ	<LOQ	<LOQ	502.8 ± 2.3	<LOQ	<LOQ	991.2 ± 12.1	<LOQ	<LOQ	<LOQ
18	Diosmetin-*C*-hexoside-*O*-deoxyhexoside	<LOQ	<LOQ	38.1 ± 0.7	<LOQ	<LOQ	<LOQ	<LOQ	<LOQ	<LOQ	<LOQ
19	Diosmetin-*C*-hexoside	<LOQ	<LOQ	<LOQ	<LOQ	<LOQ	<LOQ	<LOQ	<LOQ	<LOQ	<LOQ
20	Luteolin-*O*-malonyl-*C*-hexoside (1)	<LOQ	<LOQ	<LOQ	318.8 ± 1.4	251.0 ± 59.2	<LOQ	<LOQ	<LOQ	457.8 ± 11.1	444.2 ± 4.5
21	Luteolin-*O*-malonyl-*C*-hexoside (2)	<LOQ	<LOQ	<LOQ	1486.3 ± 14.2	651.1 ± 17.0	<LOQ	1622.9 ± 16.7	<LOQ	426.8 ± 3.4	631.3 ± 24.1
22	Apigenin-*O*-malonyl-C-hexoside (1)	<LOQ	<LOQ	<LOQ	<LOQ	<LOQ	<LOQ	<LOQ	<LOQ	<LOQ	<LOQ
23	Apigenin-*O*-deoxyhexoside-*O*-malonyl-*C*-hexoside (1)	<LOQ	<LOQ	<LOQ	<LOQ	<LOQ	<LOQ	<LOQ	<LOQ	<LOQ	172.1 ± 12.0
24	Apigenin-*O*-malonyl-*C*-hexoside (2)	<LOQ	<LOQ	<LOQ	<LOQ	<LOQ	<LOQ	<LOQ	124.9 ± 1.7	<LOQ	<LOQ
25	Apigenin-*O*-deoxyhexoside-*O*-malonyl-*C*-hexoside (2)	<LOQ	<LOQ	<LOQ	657.8 ± 6.7	171.3 ± 4.5	<LOQ	<LOQ	<LOQ	<LOQ	141.4 ± 6.2
26	Apigenin-*O*-malonyl-*C*-hexoside (2)	<LOQ	<LOQ	<LOQ	<LOQ	<LOQ	<LOQ	696.2 ± 4.1	<LOQ	148.4 ± 12.4	614.9 ± 23.4
	***Flavonoids O-glycosides and di-O-glycosides***										
27	Quercetin *O*-hexoside-*O*-deoxyhexoside	<LOQ	<LOQ	<LOQ	939.7 ± 18.9	<LOQ	<LOQ	<LOQ	2988.5 ± 180.1	<LOQ	<LOQ
28	Rutin	72.9 ± 3.4	112.9 ± 3.4	<LOQ	681.1 ± 9.7	168.4 ± 3.4	<LOQ	704.7 ± 4.2	1041.2 ± 25.1	<LOQ	<LOQ
29	Quercetin *O*-hexoside (1)	<LOQ	<LOQ	<LOQ	352.1 ± 20.6	<LOQ	<LOQ	<LOQ	519.2 ± 98.6	<LOQ	<LOQ
30	Quercetin *O*-hexoside (2)	<LOQ	<LOQ	<LOQ	<LOQ	<LOQ	<LOQ	<LOQ	655.6 ± 3.8	524.8 ± 19.9	161.3 ± 4.5
31	Quercetin *O*-pentoside (1)	<LOQ	<LOQ	<LOQ	<LOQ	<LOQ	<LOQ	<LOQ	<LOQ	<LOQ	<LOQ
32	Quercetin *O*-pentoside (2)	<LOQ	<LOQ	<LOQ	<LOQ	<LOQ	<LOQ	<LOQ	423.5 ± 8.0	<LOQ	<LOQ
33	Quercetin *O*-hexoside-*O*-hexoside	<LOQ	<LOQ	<LOQ	<LOQ	<LOQ	<LOQ	<LOQ	<LOQ	<LOQ	<LOQ
	**Total Flavonoids (TF)**	**3890.4 ± 96.9**	**6986.8 ± 35.0**	**4128.0 ± 27.8**	**12455.4 ± 2.8**	**9513.3 ± 241.1**	**4994.8 ± 130.3**	**14011.7 ± 130.7**	**12386.9 ± 174.0**	**6002.7 ± 38.1**	**8247.9 ± 297.4**
	***Flavonolignans***										
34	Flavonolignan 1	202.6 ± 18.0	241.1 ± 22.0	144.1 ± 8.1	<LOQ	205.6 ± 9.0	510.2 ± 2.9	138.5 ± 13.2	98.0 ± 6.8	<LOQ	<LOQ
35	Flavonolignan 2	799.1 ±	982.5 ± 97.8	609.3 ± 27.2	280.9 ± 6.7	864.0 ± 42.8	1857.1 ± 61.8	653.0 ± 40.6	688.8 ± 44.0	<LOQ	< LOQ
36	Flavonolignan 3	<LOQ	<LOQ	<LOQ	<LOQ	<LOQ	<LOQ	57.1 ± 8.0	121.3 ± 6.0	<LOQ	< LOQ
	**Total flavonolignans**	**1001.7 ± 65.5**	**753.4 ± 33.0**	** 753.4 ± 33.0**	**280.9 ±6.7**	**1069.6 ± 50.6**	**2367.3 ± 63.0**	**848.7 ± 51.7**	**908.1 ± 44.7**	**<LOQ**	**<LOQ**
	**Compounds**	**CP-3**	**CP-4**	**CP-C**	**CI-1**	**CI-2**	**CI-3**	**CH-1**	**CH-2**	**CHO-C**	
	***Phenolic acids***										
1	Chlorogenic acid	836.3 ± 12.6	927.5 ± 18.2	78.7 ± 4.3	1644.1 ± 44.7	323.4 ± 12.8	1331.8 ± 16.1	2881.9 ± 74.0	993.4 ± 110.6	1492.6 ± 8.3	
	***Flavonoids C-glycosides and di-C***,***O-glycosides***										
2	Luteolin *C*-hexoside-*O*-hexoside (1)	<LOQ	<LOQ	<LOQ	<LOQ	<LOQ	<LOQ	<LOQ	<LOQ	<LOQ	
3	Isoorientin-2′′-*O*-xyloside	<LOQ	<LOQ	<LOQ	1034.2 ± 5.8	838.7 ± 38.5	2066.8 ± 28.8	<LOQ	<LOQ	53.5 ± 1.6	
4	Luteolin *C*-hexoside-*O*-hexoside (2)	<LOQ	<LOQ	<LOQ	<LOQ	48.1 ± 7.9	<LOQ	<LOQ	<LOQ	295.0 ± 3.6	
5	Isoorientin-4′′-*O*-xyloside	<LOQ	<LOQ	22.4 ± 1.2	67.2 ± 8.2	43.0 ± 4.3	112.4 ± 1.6	<LOQ	<LOQ	755.4 ± 21.1	
6	Isoorientin	1066.0 ± 22.3	1993.5 ± 20.3	187.7 ± 1.2	491.7 ± 4.0	652.9 ± 39.9	2487.9 ± 32.4	444.2 ± 8.1	882.0 ± 96.5	1085.9 ± 15.9	
7	Orientin	926.6 ± 15.2	1562.5 ± 19.4	24.1 ± 0.9	<LOQ	<LOQ	<LOQ	<LOQ	<LOQ	337.7 ± 5.2	
8	Isoorientin-2′′-*O*-rhamnoside	<LOQ	<LOQ	<LOQ	82.5 ± 1.7	84.4 ± 9.6	<LOQ	<LOQ	<LOQ	<LOQ	
9	Luteolin *C*-hexoside-*O*-pentoside	<LOQ	<LOQ	<LOQ	93.6 ± 4.5	59.1 ± 7.4	309.4 ± 9.3	<LOQ	<LOQ	79.1 ± 1.1	
10	Isovitexin-2′′-*O*-glucoside	<LOQ	<LOQ	<LOQ	86.9 ± 2.0	47.9 ± 1.7	<LOQ	<LOQ	<LOQ	146.8 ± 6.4	
11	Isovitexin-2′′-*O*-xyloside	<LOQ	231.2 ± 7.1	26.9 ± 0.6	3213.6 ± 61.0	2416.4 ± 143.3	1677.5 ± 23.8	<LOQ	<LOQ	390.3 ± 7.7	
12	Vitexin	625.2 ± 2.5	457.3 ± 2.7	<LOQ	<LOQ	<LOQ	<LOQ	<LOQ	<LOQ	<LOQ	
13	Apigenin *C*-hexoside-*O*-pentoside	<LOQ	<LOQ	42.4 ± 2.6	110.5 ± 17.1	79.5 ± 13.3	<LOQ	<LOQ	<LOQ	<LOQ	
14	Diosmetin *C*-hexoside-*O*-pentoside	<LOQ	<LOQ	<LOQ	73.0 ± 1.1	102.1 ± 5.9	237.0 ± 4.3	<LOQ	<LOQ	506.7 ± 6.2	
15	Isovitexin 2′′-*O*-rhamnoside	3379.7 ± 51.4	2132.5 ± 25.3	<LOQ	587.1 ± 7.2	672.4 ± 18.2	<LOQ	<LOQ	<LOQ	<LOQ	
16	Apigenin *C*-hexoside-*O*-xyloside	<LOQ	<LOQ	<LOQ	329.4 ± 8.4	219.5 ± 21.0	<LOQ	<LOQ	<LOQ	<LOQ	
17	Isovitexin	<LOQ	<LOQ	121.2 ± 1.3	<LOQ	<LOQ	795.6 ± 12.5	<LOQ	<LOQ	<LOQ	
18	Diosmetin-*C*-hexoside-*O*-deoxyhexoside	<LOQ	<LOQ	<LOQ	<LOQ	<LOQ	<LOQ	<LOQ	<LOQ	<LOQ	
19	Diosmetin-*C*-hexoside	338.3 ± 14.2	<LOQ	21.2 ± 0.6	<LOQ	<LOQ	<LOQ	<LOQ	<LOQ	<LOQ	
20	Luteolin-*O*-malonyl-*C*-hexoside (1)	<LOQ	540.9 ± 3.4	<LOQ	<LOQ	<LOQ	<LOQ	<LOQ	<LOQ	<LOQ	
21	Luteolin-*O*-malonyl-*C*-hexoside (2)	314.0 ± 31.6	332.4 ± 3.9	<LOQ	<LOQ	<LOQ	<LOQ	144.9 ± 3.6	173.6 ± 20.7	<LOQ	
22	Apigenin-*O*-malonyl-C-hexoside (1)	244.2 ± 69.0	70.4 ± 0.4	<LOQ	<LOQ	<LOQ	<LOQ	<LOQ	<LOQ	<LOQ	
23	Apigenin-*O*-deoxyhexoside-*O*-malonyl-*C*-hexoside (1)	<LOQ	<LOQ	<LOQ	<LOQ	<LOQ	<LOQ	<LOQ	<LOQ	<LOQ	
24	Apigenin-*O*-malonyl-*C*-hexoside (2)	<LOQ	<LOQ	<LOQ	<LOQ	<LOQ	<LOQ	<LOQ	<LOQ	<LOQ	
25	Apigenin-*O*-deoxyhexoside-*O*-malonyl-*C*-hexoside (2)	196.3 ± 2.5	58.5 ± 1.5	<LOQ	<LOQ	<LOQ	<LOQ	<LOQ	<LOQ	<LOQ	
26	Apigenin-*O*-malonyl-*C*-hexoside (2)	478.5 ± 5.4	116.6 ± 1.1	<LOQ	<LOQ	<LOQ	<LOQ	<LOQ	<LOQ	<LOQ	
	***Flavonoids O-glycosides and di-O-glycosides***										
27	Quercetin *O*-hexoside-*O*-deoxyhexoside	<LOQ	<LOQ	<LOQ	<LOQ	<LOQ	<LOQ	2884.2 ± 102.9	1491.5 ± 171.0	<LOQ	
28	Rutin	<LOQ	<LOQ	53.5 ± 1.2	<LOQ	<LOQ	<LOQ	3949.2 ± 116.7	2105.6 ± 218.6	<LOQ	
29	Quercetin *O*-hexoside (1)	<LOQ	294.9 ± 9.0	<LOQ	127.4 ± 2.8	93.3 ± 2.5	800.7 ± 21.2	1388.9 ± 37.0	1105.2 ± 121.7	<LOQ	
30	Quercetin *O*-hexoside (2)	<LOQ	<LOQ	23.9 ± 0.8	453.0 ± 8.2	248.1 ± 5.0	825.8 ± 28.4	4146.0 ± 144.3	3957.0 ± 43.0	<LOQ	
31	Quercetin *O*-pentoside (1)	<LOQ	<LOQ	<LOQ	<LOQ	<LOQ	<LOQ	226.9 ± 7.5	165.5 ± 18.4	<LOQ	
32	Quercetin *O*-pentoside (2)	<LOQ	<LOQ	<LOQ	<LOQ	<LOQ	215.7 ± 8.1	1474.8 ± 47.9	1040.1 ± 115.2	<LOQ	
33	Quercetin *O*-hexoside-*O*-hexoside	<LOQ	<LOQ	<LOQ	<LOQ	<LOQ	<LOQ	240.1 ± 17.1	179.1 ± 25.3	<LOQ	
	**Total Flavonoids (TF)**	**7568.8 ± 69.8**	**7790.8 ± 44.9**	**523.3 ± 5.9**	**6750.1 ± 99.9**	**5605.4 ± 275.6**	**9528.8 ± 114.2**	**14899.2 ± 470.9**	**11099.6 ± 702.3**	**3650.5 ± 50.5**	
	***Flavonolignans***										
34	Flavonolignan 1	<LOQ	<LOQ	<LOQ	<LOQ	<LOQ	<LOQ	<LOQ	<LOQ	<LOQ	
35	Flavonolignan 2	260.3 ± 17.1	<LOQ	<LOQ	<LOQ	<LOQ	<LOQ	<LOQ	<LOQ	<LOQ	
36	Flavonolignan 3	<LOQ	213.9 ± 8.0	<LOQ	179.1 ± 4.5	227.8 ± 20.3	<LOQ	<LOQ	<LOQ	<LOQ	
	**Total**	**260.3 ± 17.1**	**213.9 ± 8.0**	**<LOQ**	**179.1 ± 4.5**	**227.8 ± 20.3**	**<LOQ**	**<LOQ**	**<LOQ**	**<LOQ**	

CO, CP, CI and CH correspond to authentic leaves of *C*. *obstusifolia*, *C*. *peltata*, *C*. *insignis* and *C*. *hispidissima* samples (see supplementary Fig. S1). CO-C, CP-C and CHO-C correspond to commercial products of *C*. *obstusifolia*, *C*. *peltata* and *C hololeuca*. Contents of analytes are reported as mean ± standard deviation (n = 3). Content below the limit of quantification: <LOQ.

Although a broad variety of flavonoid glycosides was detected, most flavonoids belonged to the flavone or flavonol classes and that the main skeletons were derivatives of luteolin, apigenin or quercetin. Among all samples, flavone *C*-glycosides were the main flavonoids subclass present in *C. obtusifolia*, *C. peltata*, *C. insignis* and *C. hololeuca*, but flavonol *O*-glycosides in *C. hispidissima*. Comparison of these results accords with those of Costa *et al*.^21^, Ortmann *et al*.^31^, da Silva Mathias and Rodrigues de Oliveira^22^ who also found that CA and flavone *C*-glycosides derived from apigenin and luteolin were the most abundant compounds detected in *C. glaziovii*, *C. pachystachya* and *C. hololeuca*.

Besides, the highest total flavonoid content was detected for *C. hispidissima* CH-1(14899.2 µg/g), followed by the *C. obtusifolia* samples: CO-7 (14071.7 µg/g), CO-4 (12455.4 µg/g) and CO-C (12387.1 µg/g). Regarding the seasonal variation of flavonoid content, a general pattern of increasing or decreasing levels among the two different periods could not be observed (See Supplemenatary Fig. S4). Similar findings were reported by Costa *et al*.^4^, who observed no correlation between the values of pluviosity and the production of *C*-glycosylflavonoids. The content of FL in *C. obtusifolia*, *C. peltata* and *C. insignis* was in the range of 179.1 (CI-1) - 2367.3 (CO-6) (µg/g). Unfortunately, it was not possible to compare the flavonolignan concentration because it was not reported previously in *Cecropia* species.

To achieve a better understanding of differences and similarities between the samples of *Cecropia* species, correlation and principal component analysis (PCA) was performed. With regard to the chemical composition, eight categories were considered: chlorogenic acid (CA), luteolin *C*-glycosydes/luteolin *C,O*-glycosydes (LG), apigenin *C*-glycosydes/apigenin *C,O*-glycosydes (AG), luteolin malonyl-*C*-glycosydes (LMG), apigenin malonyl-*C,O*-glycosydes (AMG), diosmetin *C,O*-glycosydes (DG), quercetin *O*-glycosydes (QG) and flavonolignans (FL) (see Supplementary Table S6).

Association between variables (chemical composition) was determined by the analysis of the sample correlation coefficient (See Supplementary Fig. S5). The CA level showed positive correlations with the content of LG (0.76), LMG (0.54) and AMG (0.36). Similarly, LG revealed a high association with LMG (0.72) and AMG (0.47); and FL with AG (0.40). In contrast, a negative correlation (−0.57) between AG and QG was observed. These results indicated that high concentrations of *O*-glycosides correlated with a low content of apigenin *C*-glycosides and vice-versa.

**Figure 7:**
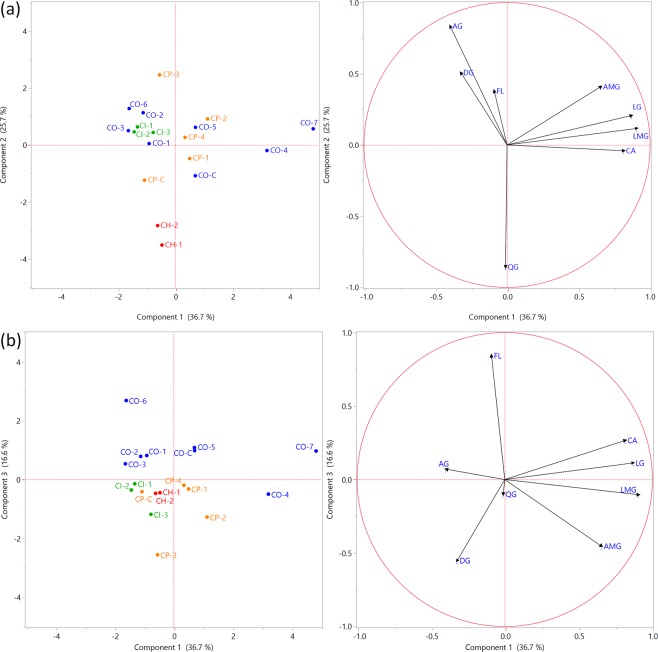
PCA score plots and loading plots of contents in *Cecropia* species, (**a**) PC1 vs. PC2 and (**b**) PC1 vs. PC3.

Two-dimensional PCA score and loading plots from Fig. 7 shows that the first three components (PC1, PC2 and PC3) accounted for 79.0 % of the cumulative variability of the original variables. The PCA results showed that *C. hispidissima* individuals (CH-1 and CH-2) were characterized by a strongly negative score on PC2. The low score of PC2 corresponds to higher values of QG and lower levels of AG. This result allowed to distinguish *C. hispidissima* from other *Cecropia* species. PC3 was able to separate *C. obtusifolia* samples (except for CO-4) from the other species. High scores of PC3 corresponded to a relatively high flavonolignans content.

## Conclusions

The present study was designed to describe the optimal experimental conditions for maximizing the extraction efficiency of leaves from plants of the genus *Cecropia* based on an experimental design and to validate an appropriate method to simultaneously quantify the polyphenolic compounds, including CA, TF and FL, in authentic and commercial samples by using high-performance liquid chromatography with diode-array detection (HPLC-DAD). Although there are some reports about analytical work from *Cecropia* species, previous investigations have not comprehensively considered the process optimization for improved phenolic compound recovery and the quantitative determination of flavonolignans in leaves from different species of this genus. Therefore, the methodology described in this work includes for the first time several parameters, such as extraction solvent, temperature and time, among others, combined with statistical tools in order to determine optimal extraction conditions and quantification of main phenolic constituents from *Cecropia*. The use of response surface methodology (RSM) was selected in this research because it can broadly be applied, due to its advantages in comparison with classical approaches (one-factor-at-a-time method), such as the capability of collecting information on many quantitative variables at once, fitting an adequate mathematical function to the experimental data and assessing the interaction effect between the parameters on the response. The results of this investigation show that solvent concentration and temperature had a significant influence on extraction, then optimization of these parameters was essential to obtain accurate and efficient recovery of the *Cecropia* leaves constituents. The optimized parameters were determined to be a temperature of 64 °C, methanol fractions (70%, v/v), extraction time of 30 min, 3 extractions a mass/solvent ratio of 1:30 (w/v) and particle size of ≤125 µm. Variations between predicted and measured recoveries below 2.0% were observed.

The HPLC–DAD method showed adequate validation parameters such as specificity, linearity, precision, accuracy, and limits of detection and quantification on ng/mL scale. It could be concluded that the HPLC–DAD method is reliable and adequate for the determination of the chemical composition of *Cecropia* samples, as an important tool for the quality control of derived commercial products. Our investigation may be useful for the discrimination of *C. obtusifolia* and *C. hispidissima* from the other species, based on their content of flavonolignans, apigenin *C*-glycosides, and quercetin *O*-glycosides.

## Methods

### Reagents

Methanol, acetonitrile, acetone, hexane (all HPLC grade), absolute ethanol and glacial acetic acid (both analytical reagent grade) were acquired from Fisher Chemical (Leicestershire, UK). Formic acid (98+%, pure, analytical reagent grade) was obtained from Acros OrganicsTM (Geel, Belgium). Ultrapure water with a resistivity of 18.2 MΩ • cm at 25 °C (Milli-Q, Waters) was used as extraction solvent and for mobile phase preparation.

Chlorogenic acid (CA) (99.0%) and rutin (RU) (96.9%) were obtained from Sigma-Aldrich (St. Louis, MO), while isovitexin (IV), orientin (OT), isoorientin (IO) (all with purity ≥99%) were from Extrasynthese (Genay, France). Vitexin (99.7%) was purchased from Adipogen (Liestal, Switzerland). All these compounds/substances were used as external standards. Flavonolignan 1, flavonolignan 2 and flavonolignan 3 (isolated from *C*. *obtusifolia* CO-1) were used as reference material.

### Plant material

The leaves of four *Cecropia* species were collected in West Panama, Panama and Chiriqui Provinces, Republic of Panama (See supplementary Fig. S1). The species were identified by the botanist Orlando O. Ortiz. The plant materials were collected in October 2015, July 2016 and July 2017. Vouchers of each collection were deposited at the Herbarium of the University of Panama

The plant material was air-dried in a general protocol oven (Heratherm^TM^, Thermo Scientific, MA, USA) at 40 °C and subsequently grounded using a mill (MF 10 Basic, IKA, Staufen, Germany). For extraction optimization and the validation of analytical methodology steps, all samples (*C*. *obtusifolia*, *C*. *peltata*, *C*. *insignis* and *C*. *hispidissima*) were pooled in equal amounts.

In addition, three commercial products were purchased online: Guarumbo Tea 200 g (Nopalife, batch No. 5000 603, México, *Cecropia obtusifolia* leaves) (CO-C), Embauba tea powder (NaturVitae, batch No. GEMPWD11123, Perú, *Cecropia peltata* leaves) (CP-C), and Umbaúba (Embaúba) leaves and stems (Chá & Cía, batch No. 052016, Brazil, *C*. *hololeuca* leaves and stems) (CHO-C).

### Sample preparation

The powdered plant material was sieved through appropriate sieves (mesh ≤710, ≤355 or ≤125 µm) and weighed in a 50 mL conical tube (VWR, Radnor, USA). Each extraction was carried out by placing the samples in an ultrasonic bath (42 kHz, 100 W) (Branson 3510, Danbury, USA). Parameter combinations were decided by a design of experiments and the extractions conducted under these designed conditions. After extraction was completed, the extract was centrifuged for 5 min at 3000 g using a Heraeus Labofuge 400 Centrifuge (Langenselbold, Germany). The supernatants were cleaned-up with an Extract CleanTM 15 mL reservoir (Grace, Deerfield, IL, USA) containing 2 g Diaion HP20 resin (Supelco, Bellefonte, PA, USA). Samples were collected in a 50.0 mL volumetric flask and subsequently diluted in a ratio 1:2 with 10% methanol. Samples were stored at −20 °C prior to analysis, if necessary.

### HPLC-DAD and HPLC-DAD-MS analysis

HPLC-DAD analysis was carried out on an Agilent 1200 series system with degasser, quaternary pump, automatic injection, thermostatic column compartment and a DAD (Agilent Technologies, Santa Clara, CA, USA). For analysis, 20 µL sample extract was injected on an RP-18 Kinetex column (2.10 × 100 mm, 2.6 μm, Phenomenex, Torrence, CA, USA). Aqueous formic acid (0.1%, v/v) and acetonitrile with 0.1% formic acid were used as mobile phases A and B, respectively. The gradient program was set as follows: 10% B (0–5 min), 10–15% B (5–20 min), 15% B (20–30 min), 15–25% B (30–40 min), 25% B (40–45 min), 25–40% B (45–55 min), 40% B (55–60 min), 40–100% B (60–65 min), 100% B (65–70 min), 100–10% B (70–75 min), 10% B (75–85 min). A flow rate of 0.7 mL/min was used. The column temperature was maintained at 26 °C. The DAD signal was recorded between 190 and 500 nm. TF and CA were monitored at 340 nm, while FL was detected at 390 nm. The analyte peak areas were used as responses for the optimization of the extraction process, while mass fraction (m/m) of CA, TF and FL from the plant material were used for the validation of the analytical method. TF was reported as the sum of all responses corresponding to flavonoids (Fig. 1).

An LC-DAD-MS system was used for the evaluation of the specificity of the HPLC-UV method and to confirm the chemical composition of the *Cecropia* species. Mass spectra were recorded using a Finnigan LXQ Mass spectrometer (Thermo Fisher Scientific, CA, USA) coupled with a Finnigan Surveyor LC system (LC Pump Plus, Autosampler Plus and PDA Plus detector). The same chromatographic conditions as described above were applied. During analysis, full scan data were recorded in ESI (−) mode from *m/z* 100 to 1800. The source, capillary and tube lens voltages; sheath and auxiliary gas flows; and the capillary temperature were set as follows: −4.0 kV, −8.0 V and −75.49 V; 65.0 L/h and 14.0 L/h; and 350 °C. DAD spectra were recorded between 200 and 400 nm. Data was analyzed using Thermo Xcalibur software (version 3.0).

### Extraction optimization

#### Ultrasound-Assisted Extraction (UAE)

An ultrasound instrument (Branson 3510, Danbury, USA) with a volume of 5.7 L, a frequency of 42 kHz and an input power of 100 W was used in the experiments. The temperature and time of extraction were monitored with a thermometer (Testo 925, Almere, Netherlands) and a timer (Fisher Scientific, CA, USA).

#### Experimental Designs

Factors affecting UAE were screened using a Fractional Factorial Design (FFD) (2^7-3^). Afterwards, based on the results of the screening step a Central Composite Design (CCD) was applied to determine the best combination of the important extraction variables^32,33^.

Fractional Factorial Design (FFD): Seven factors, two extreme levels per factor and a center point (all in duplicate) were selected. The factors were methanol fraction (MeOH %) (50 or 90%, v/v), extraction time (30 or 90 min), number of extractions with methanol (1 or 3), extraction temperature (20 or 60 °C), plant solvent ratio (1:20 or 1:100, m/v), number of extractions with acetone (0 or 2), and particle size (≤710 or ≤125 µm). FFD is shown in Supplementary Table S1.

The statistical interpretation of the estimated effect of each factor (E_x_) was performed as described by Klein-Junier *et al*.^34^. Briefly, a *t*-test was used to evaluate which factors have significant effects. The standard error of the effect *(SE)*_*e*_ was estimated based on Dong’s algorithm using the 75% lowest absolute factor effects. Unimportant factors are selected by an initial error estimation (S_0_). Finally, the critical effect (*E*_*critical*_) for the response was estimated based on (*SE*)_*e*_.

Central Composite Design (CCD): After the most important factors for extraction were determined in the screening step, variables were further optimized. Two factors at five levels were varied according to a CCD design in order to optimize the extraction process. The two factors were methanol concentration (%, v/v) (*X*_1_) and extraction temperature (°C) (*X*_2_). The factor levels with α = 1.414 are shown in Supplementary Table S2, while the design and the responses are given in supplementary Table S3.

A quadratic polynomial model used in the response surface analysis was established for each response using the following equation:

1 $$y={b}_{0}+\sum _{i=1}^{k}{b}_{i}{X}_{i}+\sum _{i=1}^{k}{b}_{ii}{X}_{i}^{2}+\sum _{i=1}\sum _{j=i+1}{b}_{ij}{X}_{i}{X}_{j}$$

where *y* is the response variable, and *b*_*0*_, *b*_*i*_, *b*_*ii*_ and *b*_*ij*_ are the regression coefficients of the model representing intercept, linear, quadratic, and interaction terms, respectively; *X*_i_ and *X*_j_ are the independent variable functions; and k the number of variables (*k* = 2).

### Validation of the analytical method

Validation studies were carried out in accordance with the guidelines of the International Conference on the Harmonization (ICH) of Technical Requirements for the Registration of Pharmaceuticals for Human Use, about the validation of analytical procedures^35^. Validation was performed in terms of specificity, linearity, precision, accuracy, limit of detection and limit of quantification.

#### Specificity

Specificity was evaluated in order to identify potential interferences by other analytes or other compounds at the chromatographic region of interest. Analytical standards (CA, VX, IV, OT, IO and RU) and sample extracts from different plant collections were analyzed to assess the selectivity of the method at the retention times of CA, flavonoids and flavonolignans within the analytical conditions established. Mass spectrometry was used to identify co-eluting substances and to determine their relevance under routine conditions. Additionally, as an indirect approach, specificity was evaluated by demonstrating acceptable accuracy (see *Accuracy*).

#### Linearity

The linearity of the method was evaluated by the calibration curves obtained by the HPLC analysis of CA, VX and RU. For the preparation of the standard solutions, a stock solution was prepared by transferring 5 mg of CA, VX and RU in a 5.0 mL volumetric flask, adding MeOH to dissolve and adjust to volume. The working standard solutions were obtained by diluting the stock solution in 10% MeOH solution to produce 0.02, 0.2, 1.6, 8.0, 20, 50, 125, 200, 350, 450, 550 and 650 µg/mL aliquots. The stock solution was stored at −20°C and the working standard solutions were prepared daily. Three determinations (n = 3) were carried out for each solution and each calibration point was fitted by linear regression. The most appropriate concentration range was determined. First-order calibration curve equations (*y* = *a* + *bx*), coefficients of determination (r^2^), residual values, 95% confidence intervals (CI) of the intercept, and t-tests (N-2, α = 0.05) for slope and intercept were calculated.

Additionally, the Mandel’s fitting test was used for mathematical verification of the first-order regression model^36^. See supplementary S. Methods.

#### Precision

The precision of the method was assessed for each analyte as repeatability (intra-day precision) and the intermediate precision (between days and concentration levels) during four different days and three concentration levels (50, 100 and 150%). Six replicates (n = 6) were performed for each working solution. The results were expressed as percentage relative standard deviation (% RSD).

The acceptability of the results was evaluated by the Horwitz equation^37,38^:

2 $${RS}{{D}}_{{R}}={2}^{(1-0.5{logC})}$$

3 $${RS}{{D}}_{{r}}=2/3[{2}^{(1-0.5{logC})}$$

where RSD_r_ is the expected coefficient of variation under repeatability conditions; RSD_R_ is the expected coefficient of variation under intermediate precision conditions; and *C* is the concentration of analyte expressed as a dimensionless mass fraction (m/m, where numerator and denominator have the same units).

#### Accuracy

Accuracy was determined by recovery (%) at three concentration levels (75, 100 and 125%) in triplicate (n = 3). The experiment was performed by adding known quantities of analytical standards (CA, VX and RU) to plant material extracts (mixture of *Cecropia* species) prepared at 50% of center point of the linear range. The recovery and 95% confidence interval (CI) were estimated.

The % RSD should be in the order of coefficient of variation for intermediate precision conditions (RSD_R_), and the recovery % is not significantly different from 100% when the 95% CI includes 100%. Additionally, a nominal range from 98 to 102% was set as recovery %.

#### Limit of detection (LoD) and limit of quantification (LoQ)

The limits of detection and of quantification were estimated based on analytical calibration curves containing the analytes (CA, VX and RU) spiked to the sample extracts. The standard deviation of the intercept was used to express the experimental error.

Additionally, these mathematical estimations of LoD and LoQ were subsequently validated by analyzing samples known to be near detection and quantification limits. Six determinations (n = 6) were performed for each concentration. Then, the lower concentrations with appropriate signal/noise ratio (S/N > 2–3.5) and precision (% RSD ≤ 5.0) were adopted as LoD and LoQ for each analyte, respectively.

### Application of the HPLC-DAD method

The proposed HPLC-DAD method was used to assess the contents of CA, TF and FL in fourteen authentic *Cecropia* leaves and three commercial products. CA, TF and FL were determined by using an external standard method, using CA (20.12 µg/mL), VX (20.32 µg/mL) and RU (18.69 µg/mL) as references. Samples were prepared in triplicate. Analytical results were reported by $\bar{x}$ ± SD, where $\bar{x}$ is the mean of results and SD is the standard deviation of measurements. Results were expressed in µg/g of dried-leaves weight.

Identity of chemical constituents was determined by comparison of their retention times with authentic reference standards or isolated compounds, and by UV/DAD spectral data. Additionally, individual flavonoids identity was confirmed by analysis of their mass spectral data.

### Statistical analysis

Data processing, calculations and graphic plotting were performed using Microsoft Office Excel 2010 (version 14.0.7), MODDE® Pro Software (version 11.0.1, MKS Umetrics, Malmö, Sweden) and GraphPad Prism for Windows (version 6.01, La Jolla, CA, USA).

The CA, TF and FL contents of *Cecropia* species were analyzed applying Principal component analysis (PCA) using the software JMP Pro 13 (SAS Institute, Cary, NC, USA).

## Supplementary information

Supplementary information
