# Supplementary material for: Ultrasound-assisted extraction optimization and validation of an HPLC-DAD method for the quantification of polyphenols in leaf extracts of Cecropia species
Source: Sci Rep. 2019 Feb 14;9:2028. doi: 10.1038/s41598-018-37607-2 (PMC6375923; doi:10.1038/s41598-018-37607-2)
Supplement: Supplementary file 1 — Supplementary information [file 41598_2018_37607_MOESM1_ESM.pdf]

Ultrasound-assisted extraction optimization and validation of an HPLC-DAD method for the quantification of polyphenols in leaf extracts of *Cecropia* species.

Andrés Rivera-Mondragón[a,*], Géraldine Broeckx[b], Sebastiaan Bijttebier[a], Tania Naessens[a], Erik Fransen[c], Filip Kiekens[b], Catherina Caballero-George[d], Yvan Vander Heyden[e], Sandra Apers[a], Luc Pieters[a], Kenn Foubert[a]

[a]Natural Products & Food Research and Analysis (NatuRA), Department of Pharmaceutical Sciences, University of Antwerp, Universiteistplein 1, 2610, Antwerp, Belgium

[b]Laboratory of Pharmaceutical Technology and Biopharmacy, Department of Pharmaceutical Sciences, University of Antwerp, Universiteitsplein 1, 2610 Antwerp, Belgium

[c]StatUa Center for Statistics, University of Antwerp, Prinsstraat 13, 2000, Belgium.

[d]Centre of Innovation and Technology Transfer, Institute of Scientific Research and High Technology Services (INDICASAT-AIP), Building 208 and 219, City of Knowledge, Panama, Republic of Panama

[e]Department of Analytical Chemistry, Applied Chemometrics and Molecular Modelling, Vrije Universiteit Brussel–VUB, Laarbeeklaan 103, B-1090 Brussels, Belgium.

*Corresponding author at:

Natural Products & Food Research and Analysis (NatuRA), Department of Pharmaceutical Sciences, University of Antwerp, Universiteitsplein 1, 2610 Antwerp, Belgium.

E-mail address: andres.riveramondragon@student.uantwerpen.be (A. Rivera).

S. Methods

HPLC-DAD and HPLC-DAD-MS analysis

Quantification was performed by an external standard method, using CA, VX and RU as reference standards for validation process. TF-1 and TF-2 were quantified as VX-1 (detected at 340 nm) or RU equivalent, respectively, while FL was expressed as VX-2 (detected at 390 nm) equivalent.

$$C = \frac{\sum A_1 \cdot\ m_2 \cdot DF_1\ \cdot\ p}{A_2 \cdot m_1 \cdot DF_2 \cdot (100 - d)} \qquad (1)$$

where C is the concentration in µg/g of CA, TF-1, TF-2 or FL; $\sum A_1$ is the area of the CA peak or the sum of the peak areas from TF-1, TF-2 or FL obtained with the test solution; A_2 is the peak area of CA, VX or RU obtained with the reference solution; m_1 is the mass of the plant material examined, in grams; m_2 is the mass of CA, VX or RU in the reference solution, in micrograms; DF_1 is the dilution factor of the test solution; DF_2 is the dilution factor of the reference solution; *p* is the percentage content of CA, VX or RU in the reference standard; *d* is the percentage lost on drying of the plant material.

Experimental Designs

Fractional Factorial Design (FFD)

The following formulas were used:

$$E_x = \frac{\sum Y(+) - \sum Y(-)}{\frac{N}{2}} \quad (2)$$

$$S_0 = 1.5 \cdot median\ |E_x| \quad (3)$$

$$(SE)_e = \sqrt{\frac{\sum E_k^2}{m}} \quad (4)$$

$$E_{critical} = t_{(\alpha,df)} \cdot (SE)_e \leftrightarrow |E_x| \quad (5)$$

where ∑Y(+) and ∑Y(-) are the sums of the responses when factor *x* is at the (+) or (–) level, respectively; N is the number of design experiments (N = 16); E_k and m are the effects and number of effects (in absolute value) ≤ 2.5 · S_0. The critical effect ($E_{critical}$) was determined with a number of degree of freedom (df) = m and α = 0.05. Values of $|E_x|$ larger or equal to $E_{critical}$ were considered significant.

Validation of the analytical method

Linearity

Mandel's fitting test

The first-order ($y = a + bx$) and the second-order ($y = a + bx + cx^2$) calibration functions, including their residual standard deviations (S_y) were determined. The difference of the variance (DS^2) and the test value (TV) were calculated for the F-test as follows:

$$DS^2 = (N-2)S_{y_1}^2 - (N-3)S_{y_2}^2,\ df = 1 \qquad (6)$$

$$TV = \frac{DS^2}{S_{y_2}^2} \qquad (7)$$

where $S_{y_1}^2$ and $S_{y_2}^2$ are the residual standard deviations from the first- and second-order calibration functions, respectively; N is the number of calibration points.

TV was compared with the value obtained from an F table (f_1 = 1, f_2 = N – 3, P = 99%). If *TV* ≤ F, the first-order calibration function provides no significant lack of fit and the second-order calibration function is not needed.

Precision

Relative standard deviation was calculated as follows:

$$RSD\ (\%) = \left({}^{S}/_{\bar{x}} \right) \cdot 100 \qquad (8)$$

where *S* is the standard deviation and $\bar{x}$ is the arithmetic mean of the measurements.

The homogeneity of variance (homoscedasticity) between different days and concentration levels was calculated by means of a Cochran's C test:

$$C = \frac{S_{max}^2}{\sum_{i=1}^{N} S_i^2} \qquad (9)$$

where *C* is the Cochran's C statistic value for the data series; S_{max}^2 is the maximum variance of the data series; S_i^2 are the variances from all data series. C values were compared with C critical values for Cochran's C at the 95% level of confidence.

Accuracy

The recovery and 95% confidence interval (CI) were calculated as follows:

$$Recovery\ \% = \frac{X_{aft\ spiking} - X_{bef\ spiking}}{X_{added}}\ x\ 100 \qquad (10)$$

$$95\%\ CI = \bar{x}_{Rec(\%)} \pm S\ x\ [\frac{t_{(1-\alpha/2,\ n-1)}}{\sqrt{n}}] \qquad (11)$$

where $X_{bef\ spiking}$ and $X_{aft\ spiking}$ are the quantity of analyte in plant material extracts before and after the analytical standard is added, respectively; X_{added} is the quantity of added analytical standard; $\bar{x}_{Rec(\%)}$ is the sample mean; S is the sample standard deviation; $t_{(1-\alpha/2,\ n-1)}$ is t-value for 95 % confidence with n-1 degree of freedom; and n is the number of samples.

Limit of detection (LoD) and limit of quantification (LoQ)

The limits of detection and of quantification were estimated based on analytical calibration curves containing the analytes (CA, VX and RU) spiked to the sample extracts. According to the following equations:

$$LoD = \frac{3.3\ \sigma}{S} \qquad (12)$$

$$LoQ = \frac{10\ \sigma}{S} \qquad (13)$$

where $\boldsymbol{\sigma}$ and S are the standard deviation of intercept and slope of the different calibration curves of analytes, respectively.

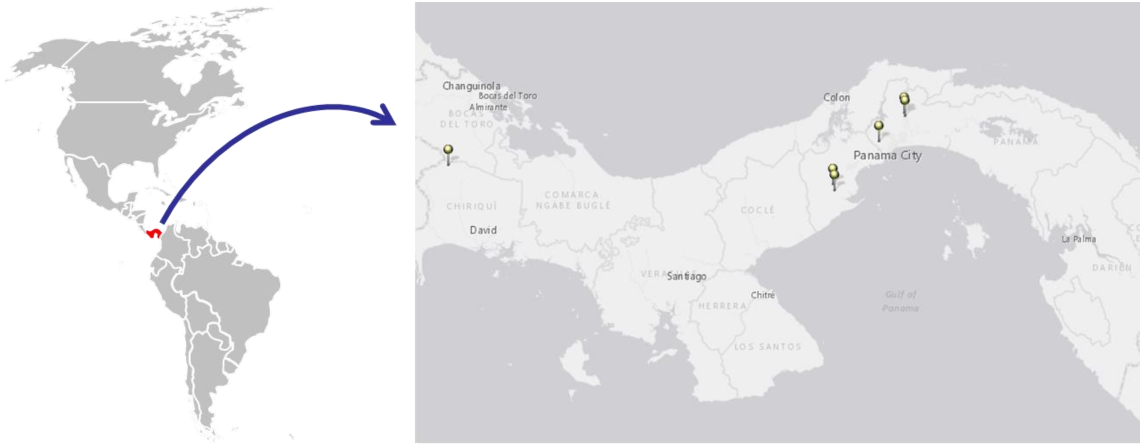

ID	Specie	Author	Voucher specimen	Province	Inflorescence	Coordenates	Date
CO-1	*C. obtusifolia*	Bertol.	2519	Panama (Cerro Azul)	Pistillate	9°12'33" N, 79°24'49" W	10/11/2015
CO-2	*C. obtusifolia*	Bertol.	2616	Panama (Cerro Azul)	Pistillate	9°11'10" N, 79°24'21" W	07/21/2016
CO-3	*C. obtusifolia*	Bertol.	2623	Panama (Cerro Azul)	Staminate	9°11'10" N, 79°24'21" W	07/21/2016
CO-4	*C. obtusifolia*	Bertol.	2527	West Panama (Cerro Campana)	Pistillate	8°41'11" N, 79°55'19" W	10/17/2015
CO-5	*C. obtusifolia*	Bertol.	2620	West Panama (Cerro Campana)	Pistillate	8°41'21" N, 79°54'55" W	07/22/2016
CO-6	*C. obtusifolia*	Bertol.	2622	Panama (Cerro Azul)	Undetermined	9°11'10" N, 79°24'21" W	07/21/2016
CO-7	*C. obtusifolia*	Bertol.	2741	Chiriquí	Staminate	8°49'32" N, 82°41'02" W	07/22/2016
CP-1	*C. peltata*	L.	2521	Panama (Camino de Cruces)	Pistillate	9°00'40" N, 79°35'44" W	10/11/2015
CP-2	*C. peltata*	L.	2625	Panama (Camino de Cruces)	Pistillate	9°00'40" N, 79°35'44" W	07/21/2016
CP-3	*C. peltata*	L.	2617	Panama (Cerro Azul)	Pistillate	9°11'10" N, 79°24'21" W	07/21/2016
CP-4	*C. peltata*	L.	2624	Panama (Cerro Azul)	Staminate	9°11'10" N, 79°24'21" W	07/21/2016
CI-1	*C. insignis*	Liebm.	2520	Panama (Cerro Azul)	Undetermined	9°11'10" N, 79°24'21" W	10/11/2015
CI-2	*C. insignis*	Liebm.	2621	Panama (Cerro Azul)	Undetermined	9°11'10" N, 79°24'21" W	07/21/2016
CI-3	*C. insignis*	Liebm.	2618	West Panama (Cerro Campana)	Undetermined	8°41'11" N, 79°55'19" W	07/22/2016
CH-1	*C. hispidissima*	Cuatrec.	2518	Panama (Cerro Azul)	Pistillate	9°11'10" N, 79°24'21" W	10/11/2015
CH-2	*C. hispidissima*	Cuatrec.	2619	Panama (Cerro Azul)	Pistillate	9°11'10" N, 79°24'21" W	07/21/2016

Figure S1. *Cecropia* samples collection points in Panama.

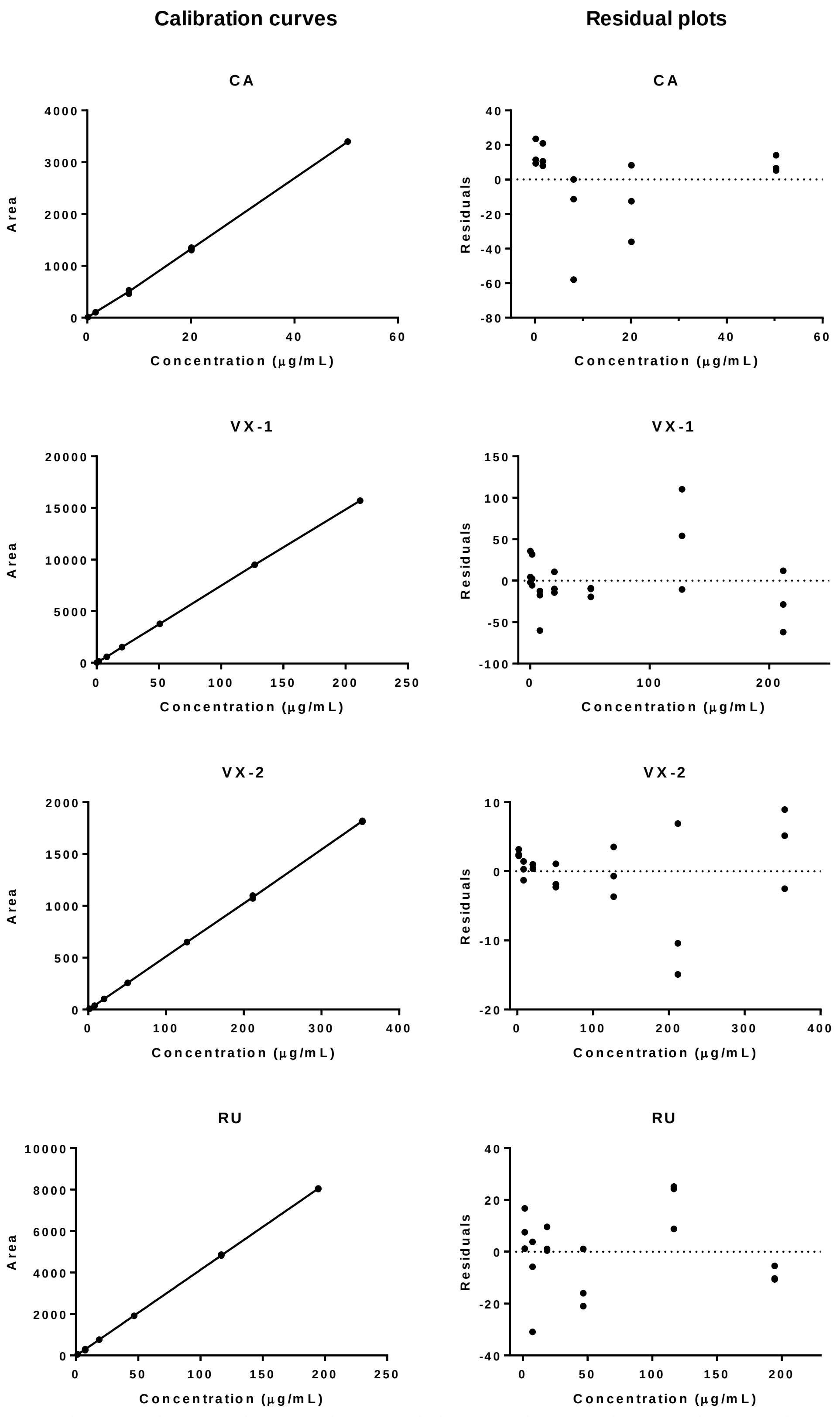

Figure S2. Calibration curves and residual plots for CA, VX-1, VX-2 and RU.

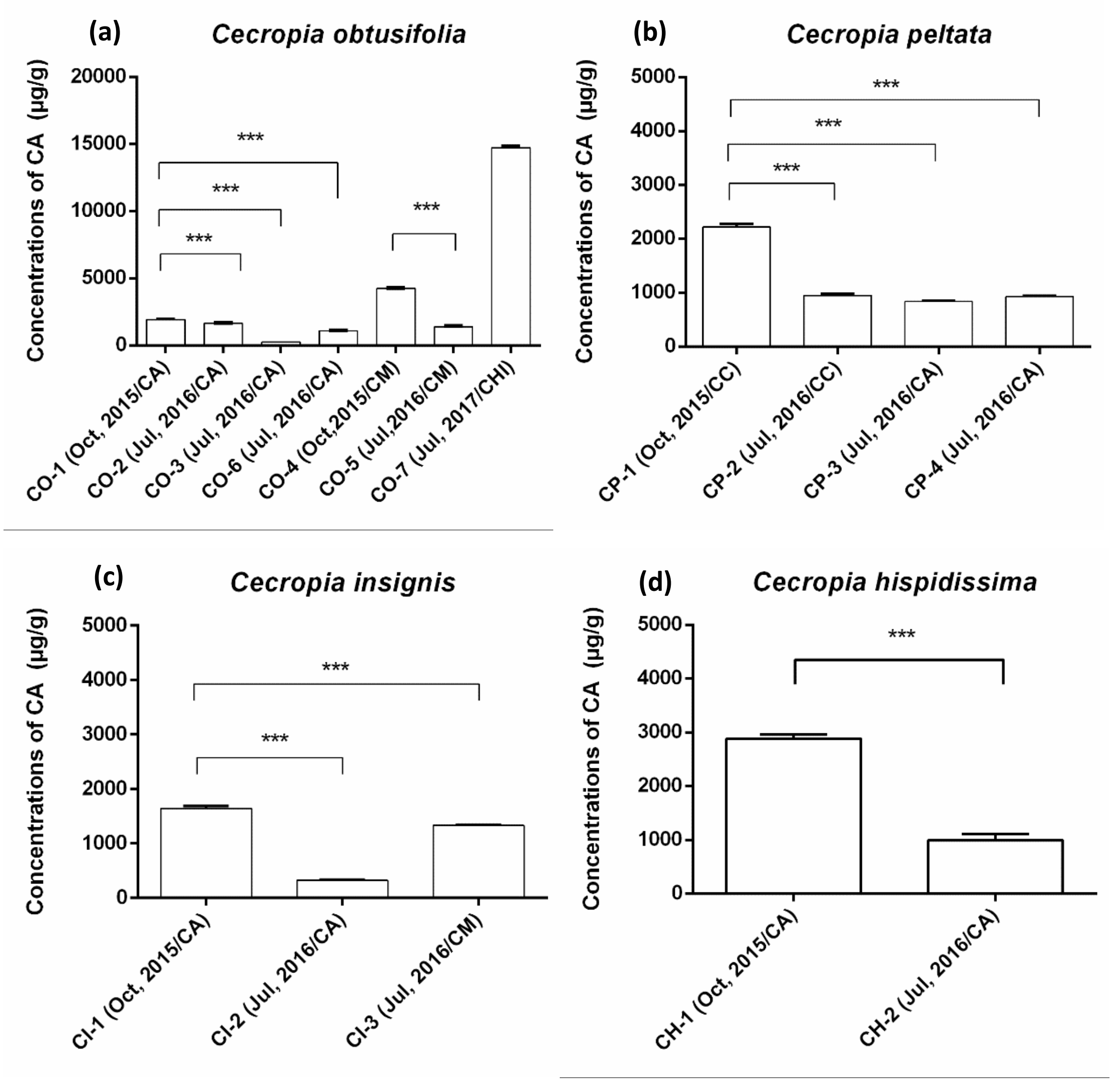

Figure S3. Concentrations of chlorogenic acid (CA) (µg/g) in authentic *Cecropia* leaf samples. (a) *Cecropia obtisofila*, (b) *Cecropia peltata*, (c) *Cecropia insignis*, and (d) *Cecropia hispidissima*. The error bar was calculated from the standard deviation (SD) of the mean. The comparison between groups was assessed by one-way analysis of variance (ANOVA) followed by Tukey test or t-test. Values of $p < 0.05$ were considered as statistically different. *** = $p < 0.001$. CA =Cerro Azul, CM = Cerro Campana, CC= Caminio de Cruces, CHI = Chiriqui.

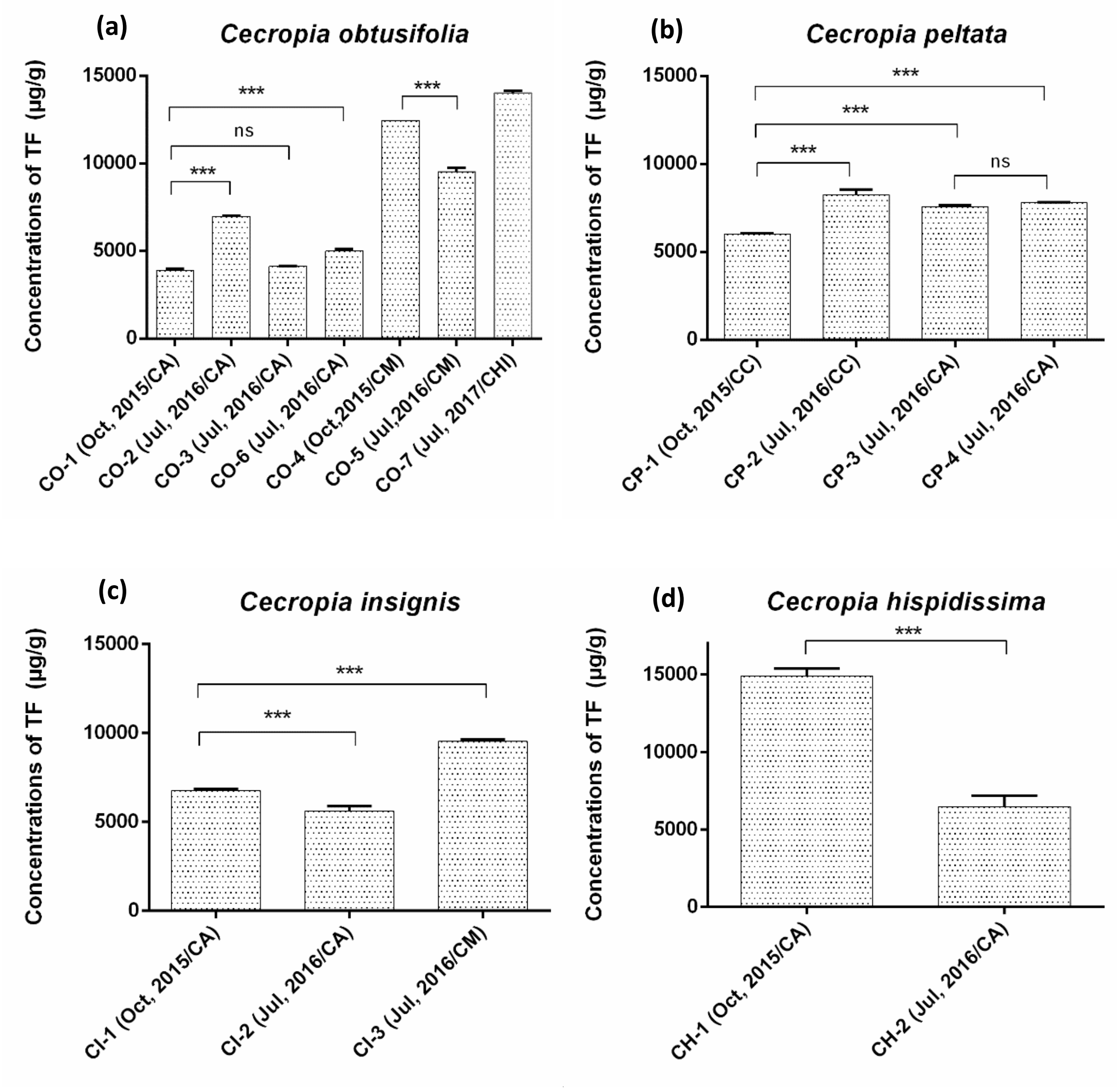

Figure S4. Concentrations of TF (total flavonoid) (µg/g) in authentic *Cecropia* leaf samples. (a) *Cecropia obtisofila*, (b) *Cecropia peltata*, (c) *Cecropia insignis*, and (d) *Cecropia hispidissima*. The error bar was calculated from the standard deviation (SD) of the mean. The comparison between groups was assessed by one-way analysis of variance (ANOVA) followed by Tukey test or t-test. Values of $p < 0.05$ were considered as statistically different. *** = $p < 0.001$, ns = no significant difference. CA =Cerro Azul, CM = Cerro Campana, CC= Caminio de Cruces, CHI = Chiriqui.

a

	CA	LG	AG	LMG	AMG	DG	QG	FL
CA	1.0000	0.7646	-0.2968	0.5445	0.3618	-0.2052	0.0901	0.1502
LG	0.7646	1.0000	-0.1690	0.7236	0.4684	-0.0963	-0.1675	0.0840
AG	-0.2968	-0.1690	1.0000	-0.1864	0.1048	0.0862	-0.5743	0.4045
LMG	0.5445	0.7236	-0.1864	1.0000	0.7116	-0.3452	-0.1328	-0.1111
AMG	0.3618	0.4684	0.1048	0.7116	1.0000	0.0023	-0.2039	-0.1511
DG	-0.2052	-0.0963	0.0862	-0.3452	0.0023	1.0000	-0.2450	-0.2330
QG	0.0901	-0.1675	-0.5743	-0.1328	-0.2039	-0.2450	1.0000	-0.2541
FL	0.1502	0.0840	0.4045	-0.1111	-0.1511	-0.2330	-0.2541	1.0000

b

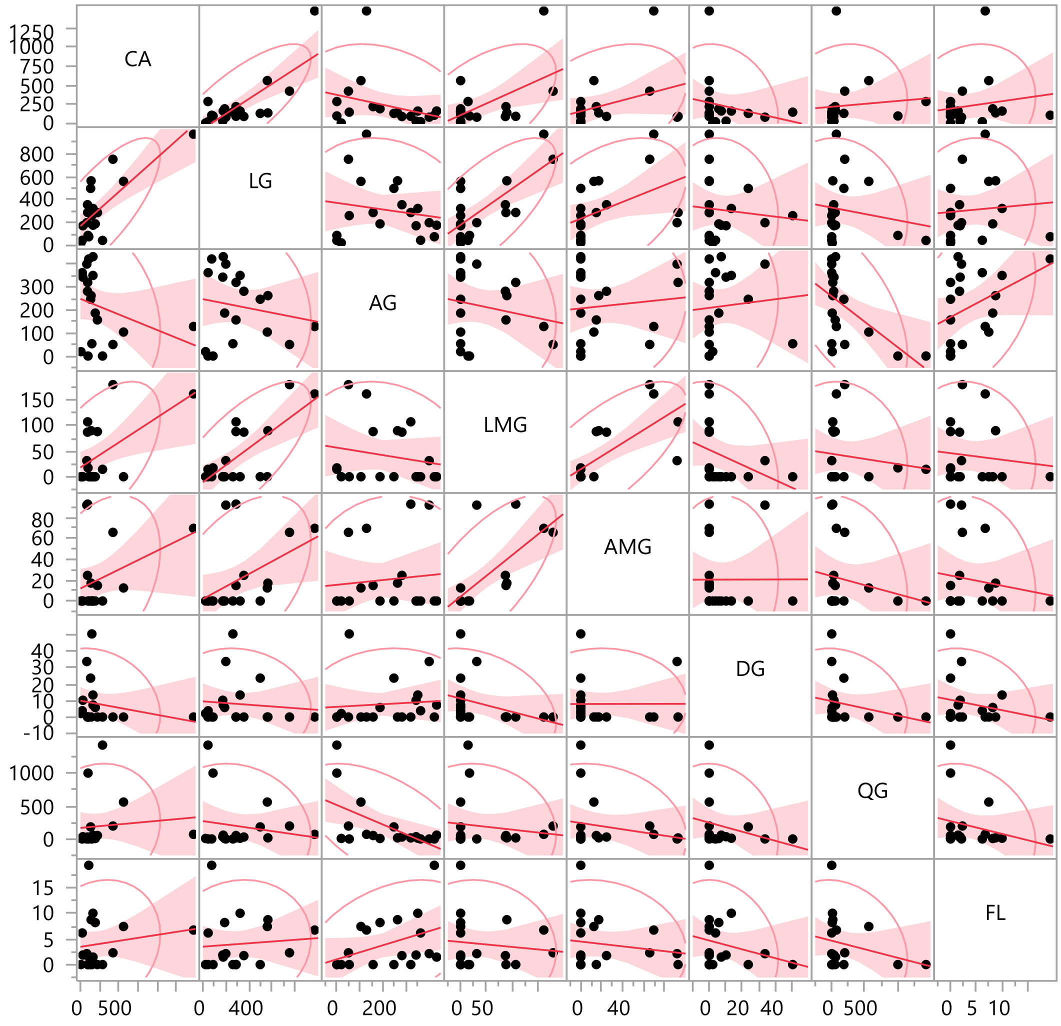

Fig. S5. Correlation analysis. a) Correlations table and b) Multivariate Scatterplot Matrix.

Table S1. Fractional factorial design (2^{7-3}) for *Cecropia* species leaves extractions. Factors: A) methanol fraction (%, v/v), B) extraction time (min), C) number of extractions, D) temperature (°C), E) mass:solvent ratio (w/v), F) number of acetone extractions, and G) particle size (µm). Responses: sum of peak areas of total flavonoids (TF), chlorogenic acid (CA) and flavonolignans (FL). The responses are represented as mean ± SD (n = 2).

**Exp. No.**	**Factors**							**Responses (Area)**		
	A	B	C	D	E	F	G	TF	CA	FL
1	50	30	1	20	20	0	≤125	1077.5 ± 37.9	199.2 ± 4.3	3.0 ± 0.01
2	90	30	1	20	100	0	≤710	902.1 ± 27.6	186.7 ± 4.2	7.9 ± 0.4
3	50	90	1	20	100	2	≤125	1480.0 ± 29.3	288.7 ± 1.2	7.5 ± 1.0
4	90	90	1	20	20	2	≤710	848.0 ± 9.9	145.9 ± 4.5	9.6 ± 0.7
5	50	30	3	20	100	2	≤710	1378.6 ± 129.4	282.6 ± 2.8	8.5 ± 1.6
6	90	30	3	20	20	2	≤125	1183.2 ± 8.6	231.8 ± 1.4	10.8 ± 0.1
7	50	90	3	20	20	0	≤710	1439.7 ± 5.6	261.0 ± 2.8	6.5 ± 0.3
8	90	90	3	20	100	0	≤125	1298.4 ± 62.9	271.8 ± 4.4	9.8 ± 1.0
9	50	30	1	60	20	2	≤710	1406.9 ± 29.2	249.0 ± 1.1	10.0 ± 0.7
10	90	30	1	60	100	2	≤125	1516.5 ± 38.3	276.7 ± 1.2	12.3 ± 0.3
11	50	90	1	60	100	0	≤710	1442.3 ± 5.7	311.7 ± 8.6	8.4 ± 0.4
12	90	90	1	60	20	0	≤125	1511.8 ± 56.1	268.1 ± 9.0	9.7 ± 1.6
13	50	30	3	60	100	0	≤125	1664.5 ± 0.9	345.1 ± 0.3	11.8 ± 0.3
14	90	30	3	60	20	0	≤710	1363.3 ± 24.1	275.3 ± 5.8	12.0 ± 0.7
15	50	90	3	60	20	2	≤125	1654.5 ± 6.2	346.4 ± 3.4	11.1 ± 0.6
16	90	90	3	60	100	2	≤710	1434.5 ± 15.8	307.2 ± 2.9	12.8 ± 0.2
17	70	60	2	40	60	1	≤355	15856.0 ± 15.5	305.1 ± 8.8	11.7 ± 0.5
18	70	60	2	40	60	1	≤355	1546.1 ± 6.1	305.1 ± 8.2	11.9 ± 0.2
19	70	60	2	40	60	1	≤355	1579.2 ± 11.5	307.2 ± 1.5	12.4 ± 0.6

Table S2. Independent variables (factors) and their levels employed in CCD for the optimization of *Cecropi*a species leaves extraction.

Coded level	Factors	
	X_1, methanol concentration (%)	X_2, extraction temperature (°C)
- α	42	36
-1	50	40
0	70	50
1	90	60
+ α	98	64

Table S3. Central composite design (CCD) for two factors and the measured responses for the optimization of the *Cecropi*a species leaves extraction. The responses are represented as mean ± SD (n = 2).

Exp. No.	Factor levels		Responses (Area)		
	*X*1	*X*2	TF	CA	FL
1	50	40	1711.8 ± 3.9	319.0 ± 4.7	12.0 ± 0.1
2	90	40	1464.8 ± 9.1	276.0 ± 6.4	13.2 ± 0.5
3	50	60	1724.5 ± 17.1	349.0 ± 1.5	12.9 ± 0.1
4	90	60	1666.8 ± 16.0	313.2 ± 5.4	13.4 ± 0.4
5	42	50	1758.5 ± 36.7	329.7 ± 2.5	12.5 ± 0.3
6	98	50	1373.6 ± 14.7	260.7 ± 3.1	13.3 ± 0.5
7	70	36	1678.3 ± 15.0	319.2 ± 4.1	13.1 ± 0.5
8	70	64	1774.1 ± 44.5	354.4 ± 3.7	14.1 ± 0.3
9	70	50	1737.8 ± 13.0	340.7 ± 0.2	13.6 ± 0.5

Table S4. Cochran's test for the determination of homogeneity of CA, TF and FL variances during 4 days and at three concentration levels. n=6, overall repeatability: intra days and levels, intermediate precision: inter days and levels. Overall variances were homogenous according Cochran's test (95% confidence level). [a] TF expressed as VX equivalent (*C.* species mixture). [b] TF expressed as rutin equivalent (*C. hispidissima*).

Analyte	Variances (S^2)						Cochran's test	
	Day (100%)				Concentration level			
	1	2	3	4	50%	150%	Test value	Critical value
CA	7.99E-07	1.57E-06	1.72E-06	6.91E-07	9.50E-07	1.11E-06	0.251	0.445
TF-1[a]	1.60E-05	3.79E-06	9.37E-06	1.46E-05	2.58E-05	1.39E-05	0.309	
TF-2[b]	9.63E-06	1.08E-05	1.65E-05	9.41E-06	1.04E-05	8.35E-06	0.253	
FL	3.52E-07	4.24E-07	6.58E-07	2.08E-07	1.94E-07	4.62E-07	0.359	

Table S5. Determination of LoD and LoQ of CA, VX and RU. Where: Limit of detection (LoD), limit of quantification (LoQ), calibration curve method (CCM), and experimental data (ED). [a]Analyte detected at 340 nm. [b]Analyte detected at 390 nm.

Analyte	LoD (ng/mL)		LoQ (ng/mL)	
	CCM	ED (S/N ratio)	CCM	ED (%RSD)
CA[a]	394.2	160.7 (3.4)	1194.6	401.9 (5.0)
VX-1[a]	346.1	131.2 (3.0)	1048.7	328.0 (5.0)
VX-2[b]	310.9	423.6 (2.1)	942.2	903.8 (2.0)
RU[a]	467.2	131.0 (2.1)	1415.9	388.6 (2.9)

Table S6.

Concentrations of chlorogenic acid (CA), luteolin *C*-glycosydes/ luteolin *C,O*-glycosydes (LG), apigenin *C*-glycosydes/ apigenin *C,O*-glycosydes (AG), luteolin malonyl-*C*-glycosydes (LMG), apigenin malonyl-*C,O*-glycosydes (AMG), diosmetin *C,O*-glycosydes (DG), quercetin *O*-glycosydes and flavonolignans (FL) (µg/g) in authentic and commercial *Cecropia* leaf samples. CO, CP, CI and CH correspond to authentic leaves of *C. obstusifolia*, *C. peltata*, *C. insignis* and *C. hispidissima* samples (see Fig. S1). CO-C, CP-C and CHO-C correspond to commercial products of *C. obstusifolia, C. peltata* and *C hololeuca.* Contents of analytes are reported as mean (n = 3). Content below the limit of quantification: <LOQ.

	**CA**	**LG**	**AG**	**LMG**	**AMG**	**DG**	**QG**	**FL**
**CO-1**	1933.2	1889.2	1871.1	<LOQ	<LOQ	58.0	72.9	82.3
**CO-2**	1634.0	3229.6	3510.1	<LOQ	<LOQ	134.1	112.9	100.6
**CO-3**	243.6	458.2	3631.6	<LOQ	<LOQ	38.1	<LOQ	61.9
**CO-4**	4238.1	7517.0	502.8	1805.1	657.8	<LOQ	1972.8	23.1
**CO-5**	1393.3	5639.8	2631.6	902.2	171.3	<LOQ	168.4	87.9
**CO-6**	1091.5	767.3	4227.5	<LOQ	<LOQ	<LOQ	<LOQ	194.6
**CO-7**	14724.5	9701.5	1286.4	1622.9	696.2	<LOQ	704.7	67.7
**CO-C**	5612.6	5590.0	1044.2	<LOQ	124.9	<LOQ	5628.0	74.6
**CP-1**	2217.8	2873.1	1571.8	884.6	148.4	<LOQ	524.8	<LOQ
**CP-2**	949.0	2877.2	3205.6	1075.6	928.4	<LOQ	161.3	<LOQ
**CP-3**	836.3	1992.5	4004.8	315.0	919.0	338.2	<LOQ	21.4
**CP-4**	927.5	3556.0	2821.0	873.3	245.5	<LOQ	294.9	17.6
**CP-C**	78.7	234.2	191.3	<LOQ	<LOQ	21.2	77.4	<LOQ
**CI-1**	1644.1	1769.3	4327.5	<LOQ	<LOQ	73.0	580.3	14.7
**CI-2**	323.4	1726.3	3435.8	<LOQ	<LOQ	102.1	341.4	18.7
**CI-3**	1331.8	4976.4	2473.1	<LOQ	<LOQ	237.0	1842.2	<LOQ
**CH-1**	2881.9	444.2	<LOQ	144.9	<LOQ	<LOQ	14310.2	<LOQ
**CH-2**	993.9	882.0	<LOQ	173.6	<LOQ	<LOQ	10044.0	<LOQ
**CHO-C**	1492.6	2606.7	537.1	<LOQ	<LOQ	506.7	<LOQ	<LOQ
